# Hexose enhances oligonucleotide delivery and exon skipping in dystrophin-deficient *mdx* mice

**DOI:** 10.1038/ncomms10981

**Published:** 2016-03-11

**Authors:** Gang Han, Ben Gu, Limin Cao, Xianjun Gao, Qingsong Wang, Yiqi Seow, Ning Zhang, Matthew J. A. Wood, HaiFang Yin

**Affiliations:** 1 Department of Cell Biology and Research Center of Basic Medical Science, Tianjin Medical University, Qixiangtai Road, Heping District, Tianjin 300070, China; 2 Molecular Engineering Laboratory, Agency for Science Technology and Research, Biomedical Sciences Institutes, 61 Biopolis Way, Singapore 138668, Singapore; 3 Department of Physiology, Anatomy and Genetics, University of Oxford, Oxford OX1 3QX, UK

## Abstract

Carbohydrate-based infusion solutions are widely used in the clinic. Here we show that co-administration of phosphorodiamidate morpholino oligomers (PMOs) with glucose enhances exon-skipping activity in Duchenne muscular dystrophy (DMD) *mdx* mice. We identify a glucose–fructose (GF) formulation that potentiates PMO activity, completely corrects aberrant *Dmd* transcripts, restores dystrophin levels in skeletal muscles and achieves functional rescue without detectable toxicity. This activity is attributed to enhancement of GF-mediated PMO uptake in the muscle. We demonstrate that PMO cellular uptake is energy dependent, and that ATP from GF metabolism contributes to enhanced cellular uptake of PMO in the muscle. Collectively, we show that GF potentiates PMO activity by replenishing cellular energy stores under energy-deficient conditions in *mdx* mice. Our findings provide mechanistic insight into hexose-mediated oligonucleotide delivery and have important implications for the development of DMD exon-skipping therapy.

## Abstract (web summary)

Exon-skipping therapies such as systemic i.v. administration of morpholino are being explored as a means of treating Duchenne muscular dystrophy. Here the authors show that adding a glucose-fructose mix can enhance uptake of phosphorodiamidate morpholino oligomer and its therapeutic effect in *mdx* mice.

## Graphical abstract

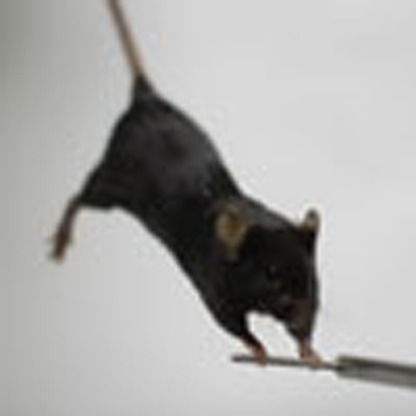

Antisense oligonucleotides (AOs) are an important class of therapeutic macromolecules with potential application in a wide range of diseases, including Duchenne muscular dystrophy (DMD)^1^,^2^,^3^. Although two lead AO chemistries for DMD are currently in clinical trials, achieving effective systemic delivery is a major challenge^2^,^3^,^4^,^5^,^6^. Phosphorodiamidate morpholino oligomer (PMO) is one class of AOs under intensive study for exon skipping therapeutic potential in DMD. Weekly intravenous administration of a 25-mer PMO^7^ to *mdx* mice, at 100 mg kg^−1^ for 7 weeks^5^ or 200 mg kg^−1^ for 12 weeks^6^, resulted in limited enhancement of dystrophin expression in skeletal muscles, with only 5–10% of wild-type levels achieved, suggesting limited systemic delivery efficiency. Several strategies, including the use of polymers and cell-penetrating peptides, have been investigated and shown to improve AO delivery efficiency to the muscle. However, their efficacy and safety profiles require further investigation before clinical application^4^,^8^,^9^, and their use may be restricted to certain AO classes; for example, direct cationic cell-penetrating peptide conjugation is typically limited to neutral-charge AO chemistries. Therefore, the development of safe and efficient delivery technologies with broad applicability is crucial to unlock the full potential of nucleic acid-based therapeutics.

Carbohydrate-based infusions, in particular 5% glucose, are widely used in clinical practice. Glucose buffers, typically 5%, are also used to improve small interfering RNA (siRNA)–exosome or siRNA–peptide complex stability for intravenous delivery^10^,^11^. For a typical intravenous injection volume (80–100 μl) of 5% glucose in a mouse with 2 ml blood, the transient rise in blood glucose concentration is ∼2–2.5 mg ml^−1^. Compared with a normal range of 0.6–1.2 mg ml^−1^ in mice with unrestricted diets^12^, the additional glucose load probably results in acute hyperglycaemia. This transient increase in blood hexose concentration might modify the physiological effects of therapy. However, this potential effect is rarely discussed or studied.

Here we show increased exon-skipping activity in *mdx* mouse muscles following co-administration of PMO^7^ with 5% glucose intramuscularly. Notably, PMO activity is further enhanced by intramuscular co-injection with other hexoses, especially a mixture of 2.5% glucose and 2.5% fructose (GF). Repeated intravenous administration of PMO in GF (PMO-GF) to *mdx* mice over 6 months increases exon skipping and induces ∼8-fold increased dystrophin protein expression without overt toxicity, compared with PMO in saline (PMO-S). The enhanced PMO activity is attributable to increased muscle uptake of PMO through an energy-dependent cellular uptake pathway, in which ATP from GF metabolism is critical. Our findings provide insight into the role of carbohydrates in oligonucleotide delivery and could inform new strategies for more effective translation of exon-skipping therapy in DMD. Moreover, by enhancing AO delivery and thereby improving the therapeutic index of nucleic acid-based therapies, the findings presented here have broad applicability.

## Results

### Carbohydrates potentiate AO exon skipping

**Figure 1:**
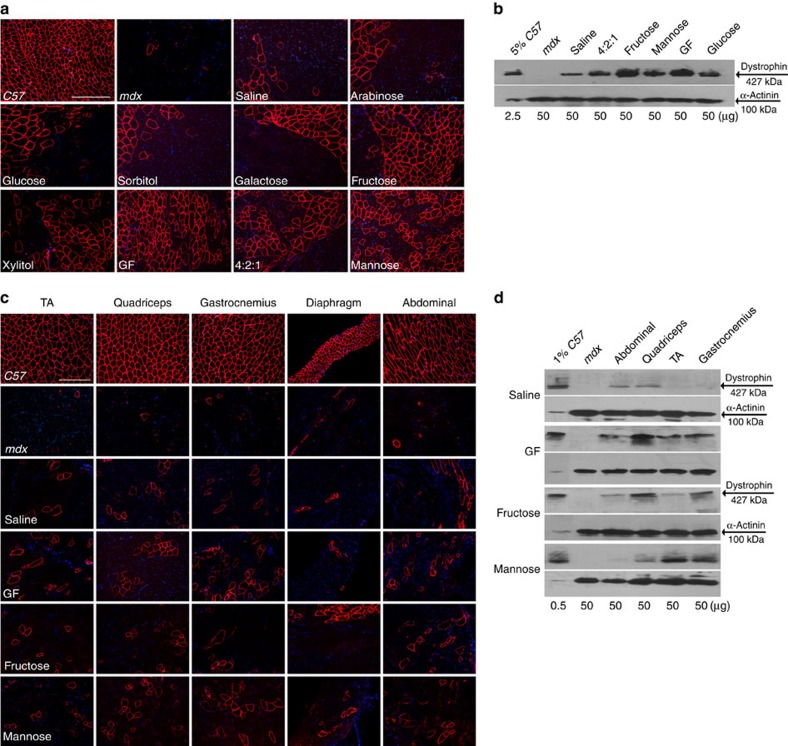
Screen of carbohydrates with PMO in *mdx* mice. Dystrophin expression following single intramuscular injection of 2 μg PMO into *mdx* TA muscles (**a**,**b**) or intravenous injection of PMO at 25 mg kg^−1^ per week for 3 weeks in different carbohydrate solutions or saline in *mdx* mice, respectively (**c**,**d**) (*n*=6). (**a**) Immunohistochemistry for dystrophin in *mdx* TA muscles. (**b**) Representative western blot image for dystrophin expression in TA muscles from different treatments. The amount of total protein loading was labelled below the image and α-actinin was used as the loading control. TA muscles from *C57BL6* were used as normal controls (the same for all western blottings unless otherwise specified). (**c**) Immunohistochemistry for dystrophin expression in body-wide muscles from *mdx* mice treated with PMO in saline or carbohydrate solutions at 25 mg kg^−1^ per week for 3 weeks intravenously. TA (scale bar, 200 μm). (**d**) Western blot analysis of body-wide muscles from *mdx* mice treated with PMO in different carbohydrates.

In initial experiments, we observed increased dystrophin protein restoration following intramuscular co-administration of PMO with 5% glucose, compared PMO with saline (PMO-S), into the tibialis anterior (TA) muscles of *mdx* mice. This was evidenced by substantial numbers of dystrophin-positive myofibres (Fig. 1a) and increased levels of dystrophin protein expression (Fig. 1b) in TA muscles treated with PMO in glucose compared with PMO-S, suggesting that glucose potentiates PMO exon-skipping activity. Given that the pathways of cellular uptake and metabolism of different hexose sugars vary, we investigated co-administration of the same PMO with other hexose analogues or combinations thereof (Table 1). Notably, co-administration of PMO with GF, 5% fructose or 5% mannose resulted in significant improvements compared with PMO-S, demonstrated by a more uniform distribution of dystrophin-positive myofibres and increased level of dystrophin restoration (Fig. 1a,b). To test this effect systemically, PMO was co-administered intravenously in GF, 5% fructose or 5% mannose in *mdx* mice at 25 mg kg^−1^ per week for 3 weeks, reasoning that differences in efficacy would be most apparent at low doses. As predicted, more uniformly distributed dystrophin-positive myofibres and higher levels of dystrophin expression were observed for PMO co-administered with hexose compared with PMO-S under identical dosing conditions (Fig. 1c,d). Up to 2% of wild-type dystrophin level was observed in quadriceps from *mdx* mice treated with PMO-GF, compared with <1% dystrophin following PMO-S treatment (Fig. 1d). The pattern of dystrophin enhancement differed in different muscle groups with fructose preferentially enhancing expression in quadriceps and gastrocnemius, and mannose preferentially enhancing expression in TA and gastrocnemius, whereas GF produced more uniform enhancement across multiple muscle groups compared with PMO-S (Fig. 1d).

Despite significant differences in dystrophin expression observed under GF, fructose and mannose conditions compared with saline, the overall level of dystrophin expression was low following low-dose PMO (25 mg kg^−1^), suggesting that higher doses are required to achieve therapeutic effects. Based on previous studies, only moderate levels of dystrophin protein were restored following repeated administration of PMO at 100 or 200 mg kg^−1^ in *mdx* mice^5^,^6^, although lower PMO doses were attempted in *mdx* mice^13^; therefore, 100–200 mg kg^−1^ was regarded as the systemic effective dose. To examine whether the application of GF could lower the systemic effective dose, a PMO dose (50 mg kg^−1^) lower than the reported effective dose but higher than 25 mg kg^−1^ was tested. PMO-GF was administered intravenously in *mdx* mice at 50 mg kg^−1^ per week for 3 weeks compared with PMO-S at 100 mg kg^−1^ per week. Repeated administration of PMO-GF at 50 mg kg^−1^ per week yielded more dystrophin-positive myofibres in TA and abdominal muscles than corresponding tissues treated with PMO-S at 100 or 50 mg kg^−1^ (Supplementary Fig. 1a). Up to 20% of wild-type dystrophin protein level was restored in abdominal muscles from *mdx* mice treated with PMO-GF at 50 mg kg^−1^ (Supplementary Fig. 1b, *n*=6), whereas ∼15 or 2.5% was restored following PMO-S treatment at 100 or 50 mg kg^−1^, respectively (Supplementary Fig. 1b, *n*=6). Overall, comparable levels of dystrophin expression were detected across different muscle groups between an effective dose of PMO-S (100 mg kg^−1^) and suboptimal dose of PMO-GF (50 mg kg^−1^). Taken together, the data indicate that GF lowers the systemic effective dose for PMO and enhances PMO-mediated exon skipping in *mdx* mice.

### PMO-GF yields long-term efficacy and functional improvement

**Figure 2:**
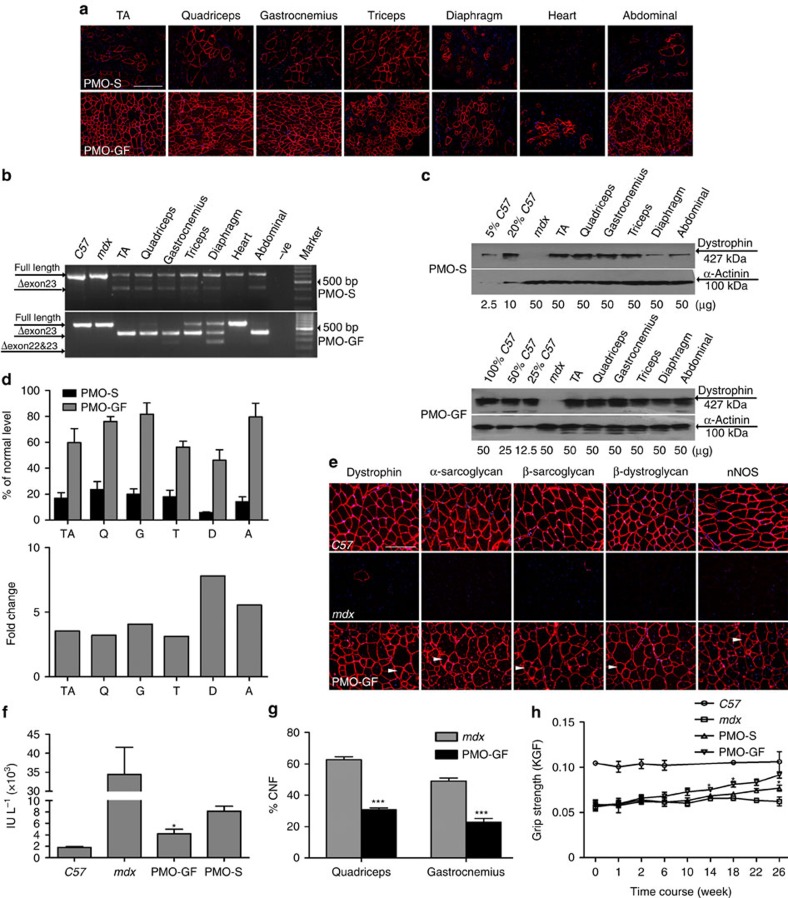
Long-term systemic efficacy of PMO-GF in *mdx* mice. Dystrophin restoration and functional improvement in *mdx* mice treated with PMO-GF at 50 mg kg^−1^ per week for 3 weeks followed by 50 mg kg^−1^ per month for 5 months intravenously. (**a**) Immunohistochemistry for dystrophin expression in body-wide muscles from *mdx* mice treated with PMO-GF (scale bar, 200 μm). (**b**) Representative RT–PCR to detect the exon-skipping efficiency, which is shown by shorter exon-skipped bands (indicated by Δexon 23, exon 23 skipped; Δexon 22&23, both exons 22 and 23 skipped). (**c**) Representative western blotting image to show dystrophin restoration in *mdx* mice treated with PMO-GF. (**d**) Quantitative analysis of western blotting results with Image J and fold change of PMO-GF relative to PMO-S. A, abdominal muscle; D, diaphragm; G, gastrocnemius; Q, quadriceps; T, triceps. Four biological replicates were examined and quantified based on densitrometry as described in Methods (*n*=4, error bars are±s.e.m). (**e**) Re-localization of DAPC components in treated *mdx* mice, to assess dystrophin function and recovery of normal myoarchitecture. The arrowheads point to identical myofibres. nNOS, neuronal nitric oxide synthase. (**f**) Measurement of serum creatine kinase (CK) levels. Data show a significant fall in *mdx* mice treated with PMO-GF compared with PMO-S and untreated age-matched *mdx* controls (*n*=6, error bars are±s.e.m; two-tailed *t*-test, *P=0.02). (**g**) Evaluation of CNFs in quadriceps and gastrocnemius from *mdx* mice treated with PMO-GF. Significant decrease was detected between PMO-GF and age-matched *mdx* controls (*n*=6, error bars are±s.e.m.; two-tailed *t*-test, ***P<0.001). (**h**) Muscle function was assessed to determine the physical improvement. Significant improvement was detected between PMO-GF and PMO-S at different time points (*n*=6, error bars are±s.e.m.; two-tailed *t* test, *P<0.05).

To determine the long-term efficacy and safety of PMO-GF, we intravenously administered PMO-GF at 50 mg kg^−1^ per week for 3 weeks followed by 50 mg kg^−1^ per month for 5 months in *mdx* mice. This resulted in substantial numbers of dystrophin-positive myofibres in multiple peripheral muscle groups, except for the heart (Fig. 2a). Near-complete exon 23 skipping was detected in quadriceps, TA, gastrocnemius and abdominal muscles, to a lesser extent in triceps and diaphragm, and no exon 23 skipping observed in the heart, from *mdx* mice treated with PMO-GF (Fig. 2b). Dystrophin was restored to near-normal levels in quadriceps, gastrocnemius and abdominal muscles treated with PMO-GF (Fig. 2c), comparable to levels obtained following biweekly injections of PMO-S at 1.5 g kg^−1^ for 6 months as reported previously^13^. Quantitative analysis of dystrophin protein revealed up to eightfold increases in diaphragm muscles treated with PMO-GF compared with muscles treated with PMO-S (Fig. 2d, *n*=6). Consistent with the molecular correction of dystrophin, pathophysiological improvement was demonstrated by re-localization of dystrophin-associated protein complex (DAPC)^14^, which fails to localize accurately to the sarcolemma in the absence of dystrophin (Fig. 2e); a significant decline in serum creatine kinase (CK) levels^15^, usually elevated in *mdx* mice (Fig. 2f, *n*=6); and a reduction in the percentage of centrally nucleated fibres in myofibres, an index of ongoing degeneration/regeneration cycles^16^ (Fig. 2g, *n*=6). Functional rescue was evident from force recovery in the grip strength test, with significant improvement following PMO-GF treatment, in particular at later time points, compared with PMO-S and untreated *mdx* controls (Fig. 2h, *n*=6). Analysis of serum biochemical indices including aspartate aminotransferase (AST), alanine aminotransferase (ALT), alkaline phosphatase, serum creatinine, urea, urinary kidney injury molecule-1 and serum glucose, and histology revealed no evidence of liver or renal toxicity, or abnormal increase in serum glucose levels (Supplementary Fig. 2a–d, *n*=6) and no inflammatory cell activation (Supplementary Fig. 2e, *n*=6). Levels of AST and ALT are elevated in *mdx* mice naturally, due to leakage from diseased muscle tissues, and thus the slightly elevated levels in treated animals unlikely reflect liver toxicity^17^. Repeated administration of PMO-GF did not lead to abnormal body-weight increase in *mdx* mice compared with those administered PMO-S and age-matched untreated controls (Supplementary Fig. 2f, *n*=6). These results demonstrate GF-mediated enhancement of PMO potency leads to remarkable functional improvements in *mdx* mice at repeated low doses without any toxicity.

### Increased nucleic acid uptake requires active transport of GF

**Figure 3:**
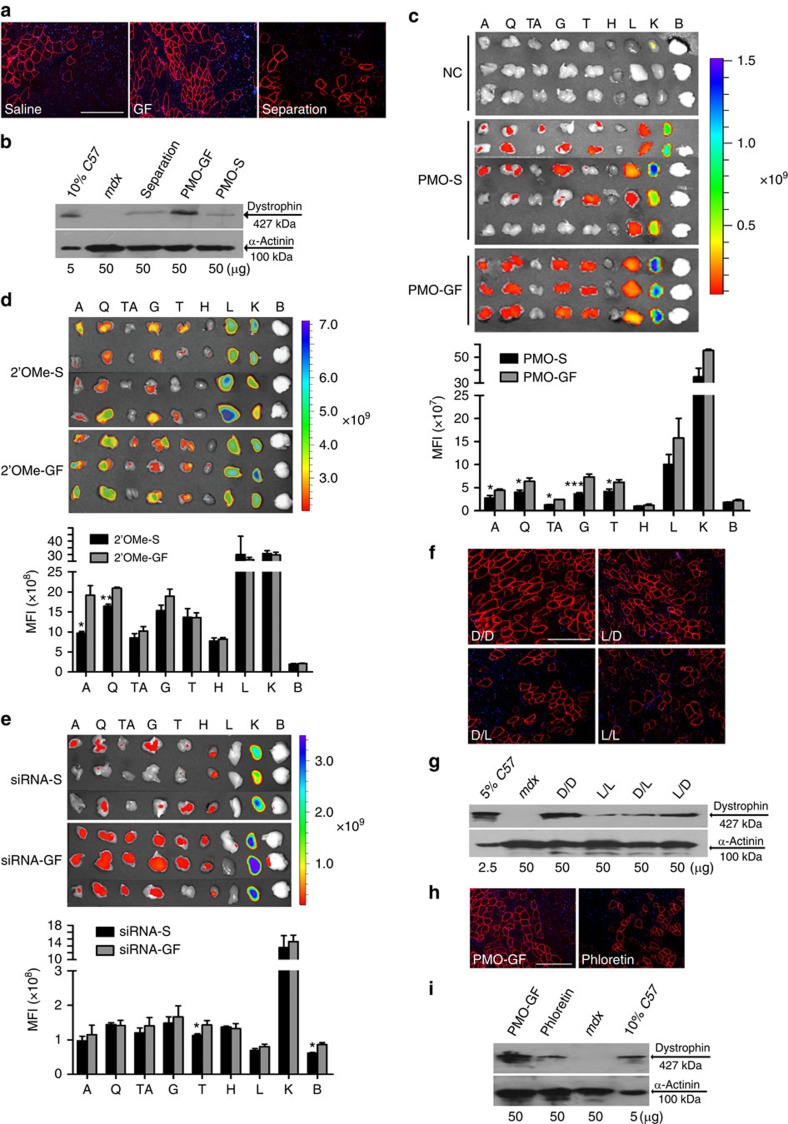
Increased muscle oligonucleotide uptake via active transport of GF. (**a**) Immunohistochemistry for dystrophin in *mdx* TA muscles injected with 2 μg PMO followed by separate administration of GF 16 h later (scale bar, 200 μm). (**b**) Western blotting to detect dystrophin protein in treated *mdx* mice. (**c**) Tissue distribution of lissamine-labelled PMO in *mdx* mice and quantitative evaluation of fluorescence intensity in body-wide tissues with IVIS spectrum series. Body-wide tissues were harvested 4 days after 3 daily intravenous injections of either PMO-GF or PMO-S at 25 mg kg^−1^ per day doses. NC represents untreated controls. A, abdominal muscle; B, brain; G, gastrocnemius; H, heart; K, kidney; L, liver; Q, quadriceps; TA, tibialis anterior; T, triceps. Significant difference was detected between PMO-GF and PMO-S for A, TA (*n*=6, error bars are±s.e.m.; Mann–Whitney *U*-test, *n*=6, *P=0.029); Q, T (*n*=6, error bars are±s.e.m.; two-tailed *t*-test, *P<0.05) and G (*n*=6, error bars are±s.e.m.; two-tailed *t*-test, ***P<0.001). (**d**) Tissue distribution of Cy5-labelled 2′OMe AOs in *mdx* mice and quantitative evaluation of fluorescence intensity in body-wide tissues. Body-wide tissues were harvested 4 days after single intravenous injection of 2′OMe in GF (2′OMe-GF) or saline (2′OMe-S) at the 100 mg kg^−1^ doses. Significant difference was detected between GF and saline in A (*n*=6, error bars are±s.e.m.; two-tailed *t*-test, *P=0.018) and Q (*n*=6, error bars are±s.e.m.; two-tailed *t*-test, **P=0.001). (**e**) Tissue distribution of Cy5-labelled siRNAs in *mdx* mice and quantitative evaluation of fluorescence intensity in body-wide tissues. Tissues were harvested 48 h after single intravenous injection of siRNAs in GF (siRNA-GF) or saline (siRNA-S) at the 8 mg kg^−1^ doses. Significant increase was detected between GF and saline in T and B (*n*=6, error bars are±s.e.m.; two-tailed *t* test, *P<0.05). (**f**) Immunohistochemistry for dystrophin expression in TA muscles of *mdx* mice 2 weeks after intramuscular injection of 2 μg PMO in different isomers of hexose (scale bar, 200 μm). (**g**) Western blotting to detect dystrophin protein in treated *mdx* mice. (**h**) Immunohistochemistry for dystrophin in TA muscles of *mdx* mice 2 weeks after intramuscular injection of 2 μg PMO in either GF or GF and 0.5 mM phloretin (scale bar, 200 μm). (**i**) Western blotting to detect dystrophin protein in treated *mdx* mice.

To determine whether the potentiating effect of GF was due to increased PMO uptake or greater exon-skipping frequency with the same intracellular PMO concentration as reported for dantrolene^18^, we administered PMO-S into TA muscles of *mdx* mice followed by injection of GF to the same muscle 16 h later, to allow for complete uptake of PMO into the muscle before GF introduction. The effect would be predicted to be similar to co-administration if GF altered the exon-skipping frequency. However, separating GF from PMO negated the enhancement observed with PMO-GF (Fig. 3a,b), suggesting that GF probably potentiates PMO activity by enhancing uptake rather than exon-skipping frequency. This notion is supported by significantly increased PMO uptake in all peripheral muscles following intravenous administration of lissamine-labelled PMO-GF compared with PMO-S (Fig. 3c, *n*=6). Notably, GF enhanced overall tissue uptake of PMO rather than altered biodistribution. Although levels of enhanced uptake varied in different tissues, peripheral muscles showed the most significant increase. Strikingly, other labelled nucleic acids including cy5-labelled 2′O-methyl phosphorothioate RNA AOs (2′OMe)^19^ or glyceraldehyde 3-phosphate dehydrogenase siRNAs^10^ displayed similar patterns of fluorescence enhancement to that of PMO, although to a lesser extent. Significant increases in mean fluorescence intensity were observed in abdominal muscles and quadriceps or triceps, and in the brain from *mdx* mice treated with either 2′OMe AOs or siRNAs in GF, respectively, compared with controls formulated in saline (Fig. 3d,e, *n*=6), suggesting that GF increases uptake of a broad range of nucleic acid therapeutics, although uptake efficiency varies.

To exclude the possibility that GF increased osmotic pressure resulting in increased membrane permeability to oligonucleotides, we compared the D-(transportable and metabolisable) and L-stereoisomers (non-transportable and non-metabolizable analogues) of component monosaccharides in GF. Strikingly, switching both glucose and fructose to L-stereoisomers completely abrogated the increased PMO exon-skipping activity, while substituting only one reduced the magnitude of the effect, indicating that active uptake and possibly metabolism of GF is critical for the enhanced AO activity. Interestingly, fructose appeared more important than glucose, as substituting L-fructose for D-fructose attenuated PMO activity to a greater degree than substituting D-glucose as determined by dystrophin protein expression (Fig. 3f,g, *n*=6). Moreover, increasing the concentration of fructose and glucose in GF induced saturation of AO-mediated exon skipping and dystrophin restoration (Supplementary Fig. 3, *n*=6), supporting the conclusion that enhanced PMO uptake was mediated by saturable active transport of GF rather than by osmotic effects. Finally, intramuscular co-administration of PMO-GF with phloretin, an inhibitor of glucose and fructose transporters^20^, abolished the potentiating effect of GF on PMO activity (Fig. 3h,i). These results suggest that active transport of GF contributes to increased uptake of PMO in the muscle.

### GF metabolism contributes to increased nucleic acid uptake

**Figure 4:**
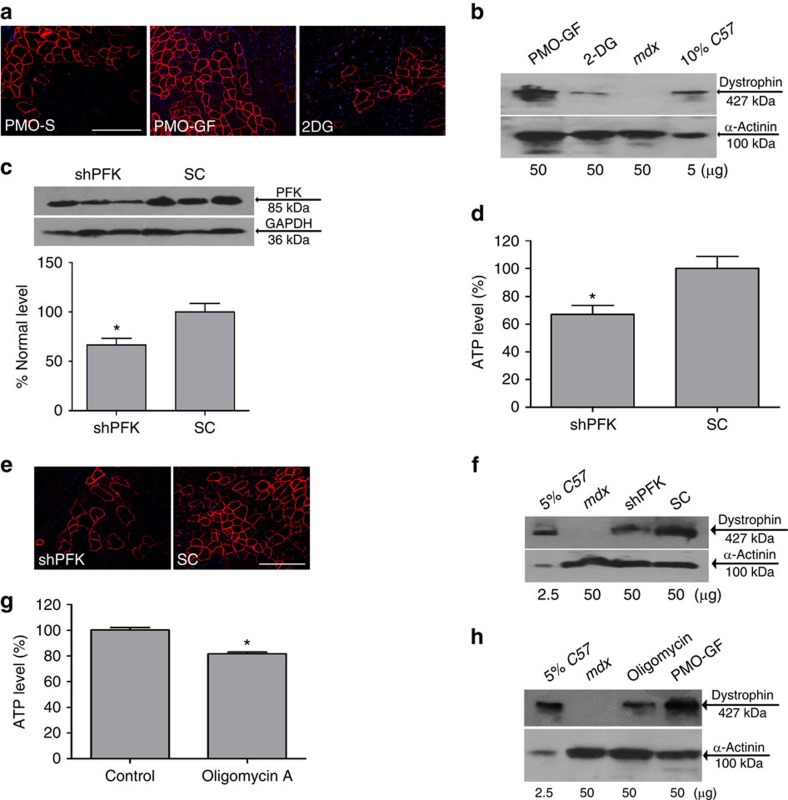
GF metabolism potentiates PMO activities in *mdx* mice. (**a**) Immunohistochemistry for dystrophin in TA muscles of *mdx* mice 2 weeks after intramuscular injections of 2 μg PMO in GF, ATP solution, 2-deoxy glucose (2DG) or both ATP and GF, respectively (scale bar, 200 μm). (**b**) Western blotting to detect dystrophin protein in treated *mdx* mice. (**c**) Western blot analysis to determine PFK1 knockdown efficiency at the protein level in TA muscles transfected with AAV-shRNA in *mdx* mice 3 weeks later. Data indicate that 41.5% of knockdown at the protein level (*n*=4, error bars are±s.e.m.; two-tailed *t* test, *P=0.024). Total protein from TA muscles treated with AAV-shRNA (30 μg) or sham control (30 μg) were loaded. Glyceraldehyde 3-phosphate dehydrogenase (GAPDH) was used as the loading control. (**d**) Measurement of ATP level in PFK1 knockdown TA muscles from *mdx* mice before administration of 2 μg PMO-GF. Data show ∼33.3% reduction at the ATP level compared with untreated *mdx* controls (*n*=4, error bars are±s.e.m.; two-tailed *t*-test, *P=0.038). (**e**) Immunohistochemistry for dystrophin-positive fibres in TA muscles either from *mdx* mice treated with AAV-shRNA or sham controls followed by intramuscular injection of 2 μg PMO-GF (scale bar, 200 μm). (**f**) Western blotting to detect dystrophin protein in treated *mdx* mice. (**g**) Measurement of ATP level in TA muscles from *mdx* mice injected with 40 ng oligomycin A before administration of 2 μg PMO-GF. Data show ∼20% reduction at the ATP level compared with untreated *mdx* controls (*n*=4, error bars are±s.e.m.; Mann–Whitney *U*-test, *P=0.029). (**h**) Western blotting to detect dystrophin protein in treated *mdx* mice.

To determine whether the potentiating effect of GF is due to enhanced co-transport of PMO with GF or to GF metabolism, we substituted GF with 2-deoxyglucose (2DG), an analogue that is actively transported and phosphorylated but cannot be further metabolized, and co-administered it with PMO into *mdx* mice. The enhancement triggered by GF was completely absent in TA muscles treated with PMO in 2DG as revealed by dystrophin restoration (Fig. 4a,b, *n*=6), indicating that GF metabolism rather than co-transport contributes to the potentiating effect. To further define the metabolic mechanism involved in GF function, we used a genetic approach to partially knock down the expression of muscle phosphofructokinase 1 (PFKm), the key enzyme regulating the rate-limiting step of hexose metabolism to generate ATP^21^. One week before administration of PMO-GF, an adeno-associated virus (AAV2/8) expressing either *PFKm* shRNA (shPFK) or a scrambled sequence control (SC) was injected locally into TA muscles of *mdx* mice. Approximately, a 41.5% reduction in PFKm protein was detected in TA muscles treated with shPFK compared with SC, resulting in a 33.3% decrease in basal ATP levels in the same muscle (Fig. 4c,d, *n*=4). This resulted in a significant decrease of dystrophin expression in shPFK-treated muscles compared with SC, following the introduction of PMO-GF (Fig. 4e,f, *n*=6), indicating that metabolism of GF is critical for the observed potentiating effect. Injection of the ATP synthase inhibitor oligomycin A^22^ alone into *mdx* TA muscles resulted in a 20% reduction in basal ATP levels (Fig. 4g, *n*=4), indicating that oligomycin A can prevent the transient rise in ATP resulting from GF metabolism. Co-administration of PMO-GF with oligomycin A resulted in a dramatic decrease in the PMO exon-skipping activity in treated TA muscles (Fig. 4h), directly implicating that ATP production promoted PMO uptake in the muscle.

### GF functions by replenishing cellular energy stores

**Figure 5:**
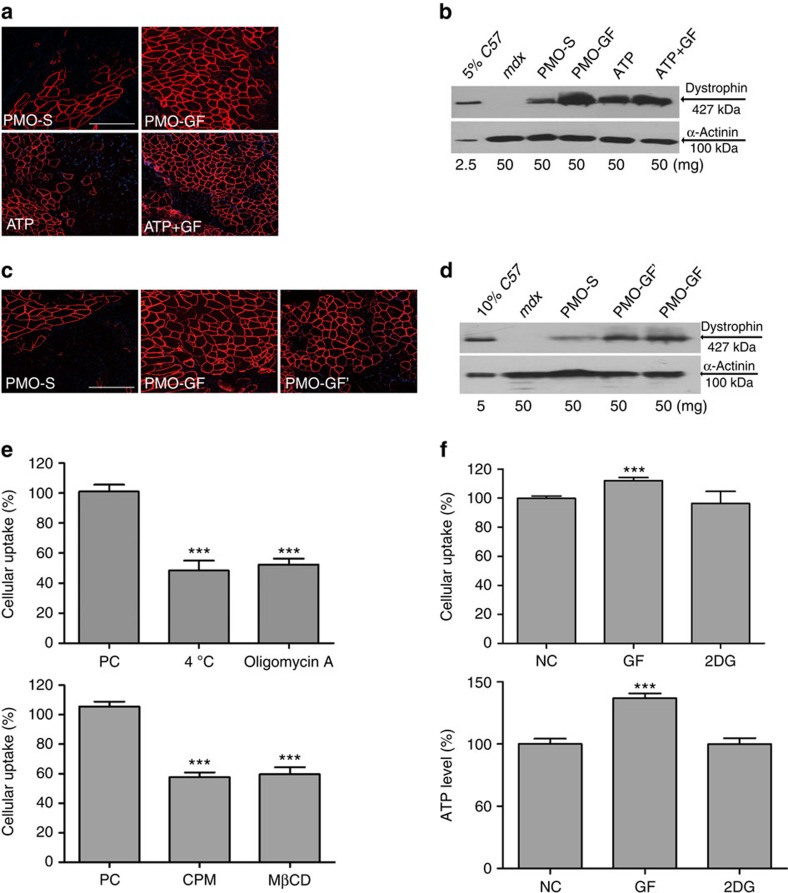
GF promotes PMO uptake by replenishing cellular stores. (**a**) Immunohistochemistry for dystrophin in TA muscles of *mdx* mice 2 weeks after intramuscular injections of 2 μg PMO in 10 mM ATP solution or both ATP and GF (scale bar, 200 μm). (**b**) Western blotting to detect dystrophin protein in treated *mdx* mice. (**c**) Immunohistochemistry for dystrophin in TA muscles of *mdx* mice 2 weeks after intramuscular injections of 2 μg PMO with simultaneous intravenous injection of GF in *mdx* mice (scale bar, 200 μm). GF' represents intramuscular injection of PMO with simultaneous intravenous injection of GF. (**d**) Western blotting to detect dystrophin protein in treated *mdx* mice. (**e**) Cellular uptake of lissamine-labelled PMO in C2C12 cells at 37 °C, 4 °C or 100 ng ml^−1^ oligomycin A suggests an energy-dependent uptake mechanism for PMO and the effect of endocytosis inhibitors on the cellular uptake of PMO in C2C12 cells. The results are shown as percentage of cellular uptake in relative to cells treated with labelled PMO at 37 °C. PC denotes 37 °C; CPM, chlorpromazine; MβCD, methyl-β-cyclodextrin (*n*=6, error bars are±s.e.m.; two-tailed *t*-test, ***P<0.001). (**f**) Cellular uptake of labelled PMO and the corresponding ATP level in starved cells before and after adding in GF or 2DG (*n*=6, error bars are±s.e.m.; two-tailed *t*-test, ***P<0.001). NC refers to starved muscle cells without the addition of GF or 2DG.

To investigate the direct role of ATP in enhancing PMO uptake in the muscle, we co-administered 10 mM ATP buffer with PMO into *mdx* mice intramuscularly, a dose equivalent to the physiological concentration in TA muscles^23^. Remarkably, increased levels of dystrophin protein expression resulted from PMO administered in ATP compared with PMO-S (Fig. 5a,b, *n*=6), suggesting that ATP directly potentiates PMO activity. To further test the hypothesis that GF functions via ATP generation, we administered PMO-GF and ATP together in *mdx* mice. A cumulative effect on PMO activity would be predicted if GF and ATP functioned through different pathways. Compared with GF alone, no synergistic increase was detected with the addition of ATP (Fig. 5a,b), suggesting that ATP and GF functioned through the same saturable pathway. Separately, we administered the same amount of PMO (2 μg) into TA muscles of *mdx* mice, followed by simultaneous intravenous injection of GF into the same *mdx* mice. An increase in dystrophin restoration was detected in TA muscles following intramuscular injection of PMO combined with intravenous injection of GF, a level similar to PMO-GF (Fig. 5c,d, *n*=6), further strengthening the conclusion that ATP from GF metabolism is responsible for the potentiating effect. It is reported that DMD patients and *mdx* mice have higher energetic requirements due to the energy-deficient status of the muscle^24^,^25^. We directly confirmed the energy-deficient status of *mdx* mouse skeletal muscles, which demonstrated significantly reduced ATP production compared with age-matched *C57BL6* controls (Supplementary Fig. 4a, *n*=6). In contrast, ATP levels were partially restored in *mdx* mouse quadriceps following repeated administration of PMO-GF at 50 mg kg^−1^ per week for 3 weeks followed by 50 mg kg^−1^ per month for 5 months (Supplementary Fig. 4b, *n*=6), supporting the conclusion that GF replenishes muscle cellular stores in *mdx* mice. Moreover, improved PMO uptake was not observed in wild-type *C57BL6* mouse muscle (that is, energetically normal muscle), whereas increased PMO uptake was detected in *SOD1G93A* mouse muscle, a model for amyotrophic lateral sclerosis, which also displays an energy-deficient status^26^ (Supplementary Fig. 5, *n*=6). Collectively, these findings support the hypothesis that GF potentiates AO activity by replenishing muscle energy stores.

To further elucidate the mechanism by which cellular energy replenishment facilitates muscle PMO uptake, we investigated the PMO uptake pathway in C2C12 cells. We first determined that PMO uptake was energy dependent as shown by reduced uptake of lissamine-labelled PMO in cells exposed to low temperature (4 °C) or ATP depletion (Fig. 5e, *n*=6). To further characterize the endocytotic uptake pathway, two well-characterized inhibitors of clathrin-dependent and -independent endocytosis were used, Chlorpromazine (CPM) and methyl-β-cyclodextrin (MβCD)^27^, respectively. Significantly reduced uptake of lissamine-labelled PMO was detected in cells treated with CPM or MβCD compared with controls (Fig. 5e), indicating that both clathrin-dependent and -independent cell uptake pathways are involved. To directly test the relationship between cellular energy replenishment generated by GF and increased PMO uptake, we modelled the energy-deficient status of *mdx* mouse muscle by starving C2C12 myoblasts for 6 h. Starvation resulted in decreased intracellular ATP levels (Fig. 5f, *n*=6 and Supplementary Fig.4); however, GF treatment significantly increased intracellular ATP levels compared with untreated, starved cells (Fig. 5f). Concurrently, a significant increase in PMO uptake was observed in starved cells treated with GF (Fig. 5f). Importantly, no increase in intracellular ATP levels or PMO uptake was detected when GF was substituted with 2DG (Fig. 5f). These findings provide direct evidence that ATP generated by GF facilitates PMO uptake under energy-deficient cellular conditions.

## Discussion

Glucose- and GF-based infusions are extensively used in clinical practice with high safety profiles. Here we demonstrate for the first time a new role for carbohydrates in facilitating nucleic acid analogue delivery to the muscle via replenishing cellular energy stores. By co-administering PMO with GF, near-normal levels of dystrophin were achieved at repeated doses of 50 mg kg^−1^, a therapeutic outcome only previously observed following biweekly PMO doses of 1.5 g kg^−1^ (ref. 13). Under identical dosing conditions, ∼8-fold increase in dystrophin expression was achieved with PMO-GF compared with PMO-S. These findings give insight into the role of carbohydrates in oligonucleotide delivery to DMD muscle and have implications for current DMD exon-skipping clinical trials, in which achieving efficient systemic delivery is a major challenge. Moreover, our data provide a more general strategy for enhancing the cellular delivery of nucleic acid analogues to the muscle and other highly energy-dependent tissues.

It is interesting to note that the combination of fructose and glucose at a 1:1 ratio performed better than either alone or other analogues. This is unexpected, as all the carbohydrates used are hexose sugar substrates that enter the glycolytic pathway to generate ATP, albeit via different pathways. This phenomenon maybe due to the glucose-dependent co-transport of fructose; for example, fructose demonstrates its greatest absorption rate in rat intestine when co-administered with glucose in equal molar ratio^28^. Moreover, co-administration with glucose enabled increased intestinal fructose absorption beyond the saturation limit demonstrated with fructose alone^29^. Thus, GF probably has synergistic effects on ATP levels required to drive active uptake of PMO. The relative selectivity of hexose transporters in muscle for different carbohydrate substrates might also affect the uptake kinetics^30^,^31^ and, consequently, the level of ATP generated.

Importantly, we did not observe any abnormal body-weight gain in *mdx* mice treated with PMO-GF, probably due to the low concentration and/or frequency of GF administered is negligible compared with dietary carbohydrates. As it is known that some DMD patients show insulin resistance and obesity^32^, close monitoring would be required when GF is translated into treatments for DMD patients in the clinic. Insulin is unlikely directly implicated in facilitating oligonucleotide uptake into the muscle as evidenced by increased dystrophin expression with PMO in 5% fructose or in ATP, both of which are not known for triggering insulin release. Moreover, plasma insulin levels after administration of GF revealed a normal physiological response of *mdx* mice (Supplementary Fig. 6), indicating no, or at most, a minor role of insulin in facilitating the PMO uptake into the muscle. It will be interesting to investigate whether the uptake of PMO declines over time with improved muscle integrity and energetics following dystrophin restoration, which is a common theoretical concern for exon-skipping therapeutics in DMD.

Intriguingly, no increase in oligonucleotide uptake in the heart was detected for *mdx* mice, suggesting energy availability is not rate limiting or, alternatively, that cardiac carbohydrate uptake and metabolism is tightly regulated. Increased dystrophin protein expression is detectable in the heart only when at least 300 mg kg^−1^ of naked PMO is administered^13^. To explore the potential of GF to enhance AO activity in the heart, we co-administered GF with R-PMO, which was previously shown to improve cardiac PMO uptake^8^. However, no improvement in heart dystrophin expression was observed at a dose of 25 mg kg^−1^ R-PMO in GF, compared with saline, suggesting that GF does not affect the uptake mechanism of peptide–PMO conjugates. As ATP flux is tightly controlled in the heart^33^, it is likely to be that a transient rise in blood carbohydrate levels does not influence energy availability in the heart. Nonetheless, additional studies on cardiac tissue are warranted with different carbohydrate formulations or modifications.

Through the sequential delineation of metabolic pathway of GF, we demonstrated that ATP from GF metabolism accounts for the functionality of GF. Importantly, we elucidated the cellular uptake pathway for PMO for the first time. An interesting observation from *in vitro* studies is that starved cells proliferated faster in the presence of GF. Therefore, it will be interesting to determine whether ATP generated by GF metabolism directly promotes the cellular uptake of PMO and/or triggers more active cell proliferation, as muscle cell regeneration was previously suggested as one of the possible mechanisms of PMO uptake in *mdx* mice^34^. Overall, the data indicate that energetic demand is a pre-requisite for the functionality of GF by compensating for the depletion of muscle energy stores under energy-deficient disease states and thus facilitating oligonucleotide uptake.

Our results demonstrate that GF can be immensely useful in nucleic acid delivery for exon-skipping therapy in DMD and potentially in other muscular dystrophies. The application of GF can significantly increase AO efficacy and/or reduce AO dose, having an impact on the probable cost of effective treatments for DMD patients. The relative ease of combining GF with nucleic acid therapeutics will accelerate the translation of nucleic acid-based therapies. Thus, our findings open a fundamentally new approach to overcome the challenge of *in-vivo* systemic delivery of therapeutic nucleic acids.

## Methods

### Animals and injections

Adult *mdx* (6–8 weeks old) and age-matched *C57BL6* mice were used in all experiments (six mice per group unless otherwise specified and equal number of male and female mice were used for each group). The mice were housed under specific pathogen-free conditions in a temperature-controlled room. The experiments were carried out in the Animal unit, Tianjin Medical University (Tianjin, China), according to procedures authorized by the institutional ethical committee (Permit Number: SYXK 2009-0001). For local intramuscular injection, 2 μg PMO was dissolved in saline or different carbohydrate solutions. For intravenous injections, various amounts of PMO in 100 μl saline or carbohydrate solution were injected into tail vein of *mdx* mice at 25, 50 or 100 mg kg^−1^ per week, respectively, for 3 weeks. For the long-term systemic study, PMO in GF or saline was repeatedly injected at 50 mg kg^−1^ per week for 3 weeks followed by 50 mg kg^−1^ per month for 5 months in *mdx* mice intravenously. Mice were killed by CO_2_ inhalation at 2 weeks after the last injection, unless otherwise specified, and muscles and other tissues were snap frozen in liquid nitrogen-cooled isopentane and stored at −80 °C.

### Oligonucleotides

PMO were synthesized by GeneTools LLC (Corvallis, Oregon, USA). Cy5-labelled 2′OMe and siRNA were purchased from TriLink BioTechnologies (San Diego, CA, USA) and Eurogentec (LIEGE Science Park, Belgium), respectively. Both PMO (5′- ggccaaacctcggcttacctgaaat -3′) and 2′OMe AO (5′- ggccaaacctcggcttacct -3′) sequences were targeted to the murine *dystrophin* exon23/intron23 boundary site as reported^7^,^20^. Glyceraldehyde 3-phosphate dehydrogenase siRNAs used were sense 5′- CAGAAGACUGU GGAUGGCC -3′ (DTDT) and antisense 5′- GGCCAUCCACAGUCUUCUG -3′ (DGDG)^11^.

### RNA extraction and nested RT–PCR analysis

Total RNA was extracted with Trizol (Invitrogen, UK) as per the manufacturer's instructions and 200 ng of RNA template was used for 20 μl reverse transcriptase–PCR (RT–PCR) with OneStep RT-PCR kit (Qiagen, UK). The primer sequences for the initial RT–PCR were Exon 20 F0: 5′- CAGAATTCTGCCAATTGCTGAG -3′ and Exon 26 R0: 5′- TTCTTCAGCTTTTGTGTCATCC -3′ for reverse transcription from messenger RNA and amplification of complementary DNA from exons 20 to 26. The cycling conditions were 95 °C for 1 min, 55 °C for 1 min and 72 °C for 2 min for 25 cycles. The primer sequences for the second rounds were Exon 20 F1: 5′- CCCAGTCTACCACCCTATCAGAGC -3′ and Exon 24 R1: 5′- CCTGCCTTTAAGGCTTCCTT -3′. The cycling conditions were 95 °C for 1 min, 57 °C for 1 min and 72 °C for 1 min for 25 cycles. The products were examined by electrophoresis on a 2% agarose gel.

### Immunohistochemistry and histology

Series of 8 μm sections were examined for dystrophin expression with a polyclonal antibody 2,166 against the dystrophin carboxy-terminal region (the antibody was kindly provided by Professor Kay Davies, University of Oxford, UK) at a dilution of 1 in 200. Polyclonal antibodies were detected by goat-anti-rabbit IgG Alexa Fluro 594 (Molecular Probe, UK). The serial sections were also stained with a panel of polyclonal and monoclonal antibodies for the detection of DAPC components. Rabbit polyclonal antibody to neuronal nitric oxide synthase (1:50) and mouse monoclonal antibodies to β-dystroglycan, α-sarcoglycan and β-sarcoglycan were used according to the manufacturer's instructions (1:200, Novocastra, UK). Polyclonal antibodies were detected by goat-anti-rabbit IgGs Alexa 594 and the monoclonal antibodies by goat-anti-mouse IgGs Alexa 594 (Molecular Probe). The M.O.M. blocking kit (Vector Laboratories, Inc., Burlingame, CA, USA) was applied for the immunostaining of the DAPC. CD3+ T lymphocytes were identified with rat polyclonal primary antibody^35^ and then detected by goat-anti-rat IgGs Alexa 594 (Molecular Probe). For macrophage staining, polyclonal rabbit primary antibody from Abcam (Cambridge, UK) was used and detected by goat-anti-rabbit IgGs Alexa 594 (Molecular Probe). Routine haematoxylin and eosin staining was used to examine the liver and kidney morphology, and assess the level of infiltrating mononuclear cells.

### Centrally nucleated fibre counts

The number of centrally nucleated muscle fibres in quadriceps and gastrocnemius muscles from *mdx* mice treated with weekly (for 3 weeks) and monthly injections (for 5 months) of PMO-GF was examined, quantified and compared with untreated age-matched *mdx* controls. Briefly, 1,000 muscle fibers were randomly selected and quantified in a double-blinded manner, with 1 or more nuclei located centrally defined as centrally nucleated fibres.

### Protein extraction and western blotting

The collected sections were placed in a 1.5-ml polypropylene Eppendorf tube on dry ice. The tissue sections were lysed with 150 μl protein extraction buffer containing 125 mM Tris-HCl pH 6.8, 10% SDS, 2 M urea, 20% glycerol and 5% 2-mercaptoethanol. The mixture was boiled for 5 min and centrifuged. The supernatant was collected and the protein concentration was quantified by Bradford assay (Sigma, USA). Various amounts of protein from normal *C57BL6* TA muscles as a positive control and from muscles of treated or untreated *mdx* mice were loaded onto SDS–PAGE gels (4% stacking and 6% resolving). Samples were electrophoresed for 4 h at 80 mA and transferred to nitrocellulose overnight at 50 V at 4 °C. The membrane was then washed and blocked with 5% skimmed milk and probed with DYS1 (monoclonal antibody against dystrophin R8 repeat, 1:200, NovoCastra) overnight. For PFKm protein assay, a mouse monoclonal antibody against a peptide mapping to the C terminus of human PFK-1 (1:1,000, Santa Cruz, Texas, USA) was used. The bound primary antibody was detected by horseradish peroxidase-conjugated rabbit anti-mouse IgGs and ECL Western Blotting Analysis system (Amersham Pharmacia Biosciences). The quantification is based on band intensity and area with Image J software, and compared with that from *C57BL6* TA muscles. Briefly, the densitometric intensity of each band, including dystrophin and α-actinin, was measured; next, the dystrophin values were divided by their respective α-actinin values. The dystrophin/α-actinin ratios of treated samples were normalized to the average *C57BL6* dystrophin/α-actinin ratios (from serial dilutions). Each experiment was performed at least three times (at least three animals). Uncropped images of all the western blottings can be found in Supplementary Fig. 7.

### Functional grip strength

Treated and control mice were tested using a commercial grip strength monitor (Chatillon, West Sussex, UK). Briefly, each mouse was held 2 cm from the base of the tail, allowed to grip a protruding metal triangle bar attached to the apparatus with their forepaws and pulled gently until they released their grip. The force exerted was recorded and five sequential tests were carried out for each mouse, averaged at 30 s apart. Subsequently, the readings for force recovery were normalized by the body weight.

### Clinical biochemistry

Serum and plasma were taken from the jugular vein immediately after killing with CO_2_ inhalation. Analysis of serum creatinine kinase (CK), alkaline phosphatase, AST, ALT, serum creatinine, urea, glucose and plasma insulin levels was performed by the clinical pathology laboratory (Tianjin Huanhu Hospital, Tianjin, China). Measurement of kidney injury molecule-1 in urine was performed with the ELISA kit as per the manufacturer's instructions (Cloud-Clone Corp., Houston, TX, USA).

### Tissue distribution

Lissamine-labelled PMO, Cy5-labelled 2′OMe and siRNAs were diluted in 100 μl of saline or GF and administered into adult *mdx* mice intravenously at 25 mg kg^−1^ per day for 3 days, and 100 and 8 mg kg^−1^ for one single injection, respectively. Mice treated with labelled PMO, 2′OMe and siRNAs were terminally anaesthetized 4 days after last injection, 4 days after single injection and 48 h after single injection, respectively. Perfusion was performed with 50 ml of cold PBS to wash out free oligonucleotides in circulation. Body-wide muscles, liver, kidney and brain were harvested for imaging and quantification with IVIS imaging system (PE, USA).

### ATP assay

The extraction of ATP from muscles was adopted and further modified from earlier protocol^24^. Briefly, muscles were harvested and snap frozen in liquid nitrogen and ∼10–20 mg of 4- to 6-μm-thick cryosections were collected into 1.5 ml Eppendorf tube. Six hundred microlitres of pre-cooled 0.4 M HClO_4_ was added in to dissolve the sections followed by vortex for 1 min on ice. The tube was spun for 5 min at 2,000 r.p.m. at 4 °C and the supernatant was transferred to a new tube and another 400 μl pre-cooled 0.4 M HClO_4_ was added into the precipitate followed by centrifugation as the previous step. Subsequently, the supernatant was spun at 4 °C for 5 min at 2,000 r.p.m. to remove debris and was stored for assay. The extraction of ATP from cells was performed as per manufacturer's instructions and CellTiter-Glo Luminescent Cell Viability Assay kit (Promega, WI, USA) was used for measuring the levels of ATP in muscles and cells.

### PFKm knockdown in *mdx* mice

For PFK1 knockdown experiment, four different short hairpin RNA (shRNA) target sequences were selected and optimized by Obio Technology Co., Ltd (Shanghai, China). PFK1 shRNA (5′- GATCCCCGGACCAGACAGACTTTGAA TTCAAGAGATTCAAAGTCTGTCTGGTCCTTTTT -3′) was selected for virus packaging in AAV2/8 serotype plasmid and sequence 5′- GATCCCC TTCTCCGAACGTGTCACGTTTCAAGAGAACGTGACACGTTCGGAGAATTTTTTGAC -3′ was designed for scrambled SC. High-titre AAV2/8 particles were produced and supplied by Obio Technology Co., Ltd. For AAV transduction, 40 μl AAV2/8 particles were injected into TA muscles of *mdx* mice and the knockdown efficiency of PFK protein was assayed at 1 and 3 weeks post injection. Two micrograms of PMO-GF was administered into the same TA muscles of *mdx* mice infected with either AAV-shPFK or AAV-SC 1 week after transduction.

### Mechanism assays

For studies of energy dependence, C2C12 cells were grown in DMEM medium (Life Technologies, USA) supplemented with 10% fetal bovine serum and 1% penicillin–streptomycin at 37 °C under 10% CO_2_. C2C12 cells were pre-incubated for 1 h at 37 °C, 4 °C or with 100 ng ml^−1^ oligomycin A (Selleck, USA). Lissamine-labelled PMO was then added at a final concentration of 20 μM and incubated for 1 h. Cells were washed with PBS solution and lysed with RIPA lysis buffer (Beyotime, China) before fluorescence was measured with a 96-well plate-reader (Nunc, USA). The maximal excitation wavelength of 570 nm and the maximal emission wavelength of 590 nm were used for measuring fluorescence.

For studies of the endocytotic pathway, C2C12 cells were grown in DMEM (Life Technologies) supplemented with 10% fetal bovine serum and 1% penicillin–streptomycin, and pre-incubated for 1 h at 37 °C in the presence of the appropriate inhibitors. Lissamine-labelled PMO was then added at the final concentration of 20 μM and further incubated for 1 h. Cells were washed and lysed as described above and measured with a 96-well plate reader. CPM (15 μM, Sigma) or MβCD (5 mM, Sigma) was used to inhibit clathrin-dependent and -independent endocytosis, respectively.

### *In vitro* rescue assay

To study the effect of GF on the PMO uptake *in vitro*, we first established a transient energy-deficient cell model by culturing C2C12 cells in glucose- and pyruvate-free DMEM with 10% serum for 6 h. The final concentration of 4.5 g l^−1^ 2′-deoxy-D-glucose (2DG) or 4.5 g l^−1^ GF together with 20 μM lissamine-labelled PMO was added into cells and incubated for 1 h, respectively. Cells were washed with PBS and lysed with RIPA buffer, and measured with the 96-well plate reader for the fluorescence intensity as described above.

### Data analysis

All data are reported as mean values±s.e.m. Statistical differences between different treated groups were evaluated by SigmaStat (Systat Software Inc., Chicago, IL, USA). Both parametric and non-parametric analyses were applied, in which the Mann–Whitney rank sum test (Mann–Whitney *U*-test) was used for samples on a non-normal distribution, whereas a two-tailed *t*-test was performed for samples with a normal distribution, respectively.

## Additional information

**How to cite this article:** Han, G. *et al*. Hexose enhances oligonucleotide delivery and exon skipping in dystrophin-deficient *mdx* mice. *Nat. Commun.* 7:10981 doi: 10.1038/ncomms10981 (2016).

## Supplementary Material

Supplementary Information Supplementary Figures 1-7.

## Figures and Tables

**Table 1:** Carbohydrates used in the study.

**Name**	**Composition**
Arabinose	5% arabinose
Glucose	5% glucose
Sorbitol	5% sorbitol
Galactose	5% galactose
Fructose	5% fructose
Xylitol	5% xylitol
GF	2.5% Glucose:2.5% fructose
4:2:1	5.7% Glucose:2.86% fructose:1.4% xylitol
Mannose	5% Mannose
