# Supplementary material for: Hexose enhances oligonucleotide delivery and exon skipping in dystrophin-deficient mdx mice
Source: Nat Commun. 2016 Mar 11;7:10981. doi: 10.1038/ncomms10981 (PMC4793046; doi:10.1038/ncomms10981)
Supplement: Supplementary Information — Supplementary Figures 1-7. [file ncomms10981-s1.pdf]

Supplementary Fig. 1

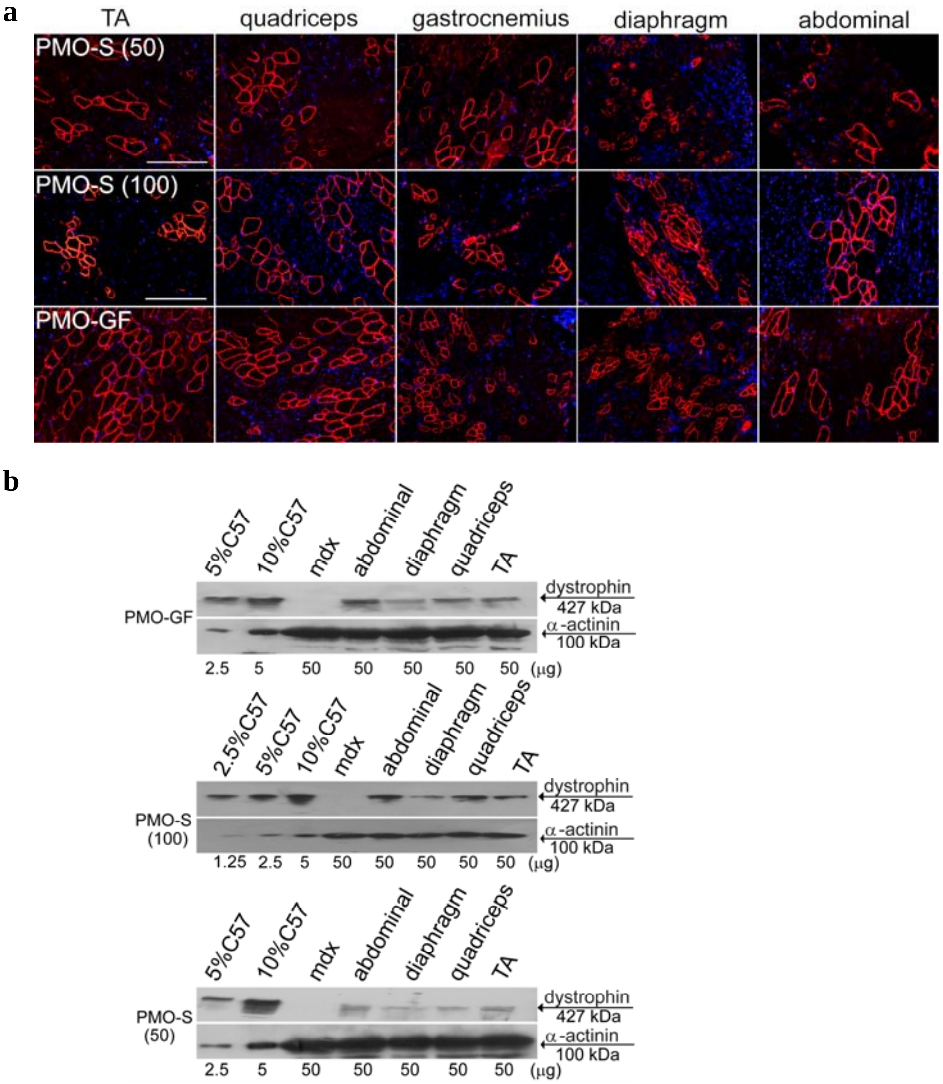

Supplementary Figure 1.

Comparison between PMO-GF and PMO-S at different doses in *mdx* mice.

(**a**) Immunohistochemistry for dystrophin in body-wide muscles of *mdx* mice treated with PMO-GF or PMO-S at 50 mg $kg^{-1}week^{-1}$ or 100 mg $kg^{-1}week^{-1}$ for 3 weeks intravenously, respectively. Samples were harvested 2 weeks after last injection (scale bar=200 μm). (50) or (100) refers to 50 or 100 mg $kg^{-1}week^{-1}$ for 3 weeks; PMO-GF was injected at 50 mg $kg^{-1}week^{-1}$ for 3 weeks.
(**b**) Western blot to detect dystrophin protein in treated *mdx* mice. Protein loading was labelled below the image and TA muscles from *C57BL6* were used as normal controls. And α-actinin was used as the loading control.

Supplementary Fig. 2

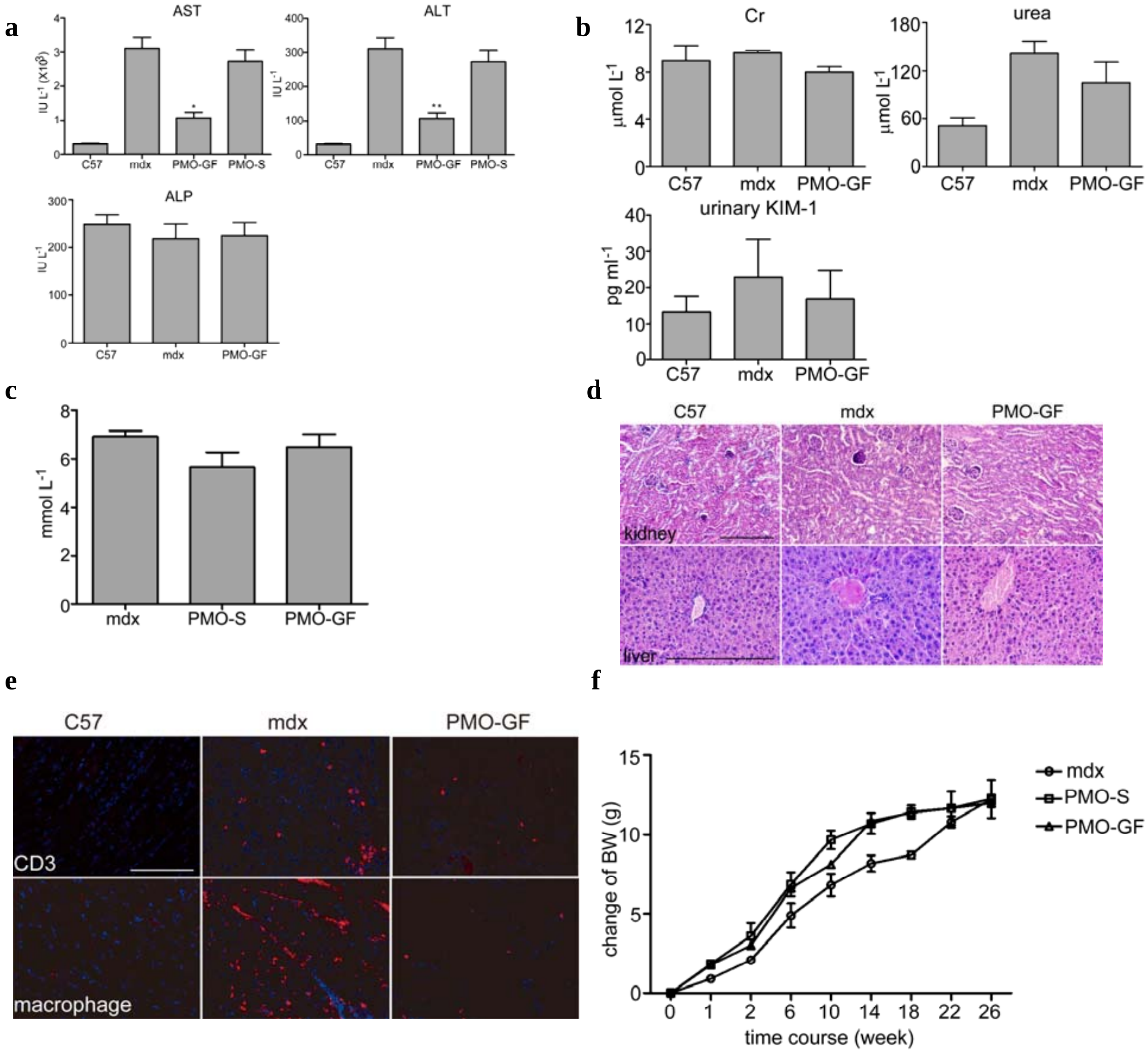

Supplementary Figure 2

Biochemical and histological measures of toxicity in PMO-GF treated *mdx* mice.

(**a**) Measurement of serum levels of liver enzymes in *mdx* mice treated with PMO-GF compared to normal and untreated *mdx* mice. Data show improved pathological parameters in *mdx* mice treated with PMO-GF compared with PMO-S and untreated *mdx* controls in levels of AST (n=6, error bars are $\pm$s.e.m; two-tailed t test, **P=0.002) and ALT (n=6, error bars are $\pm$s.e.m; two-tailed t test, *P=0.045). And no change in the level of serum alkaline phosphatase (ALP) between *mdx* mice treated with PMO-GF and untreated *mdx* controls (n=6, error bars are $\pm$s.e.m) indicated no liver toxicity elicited by the repeated administration of PMO-GF.
(**b**) Analysis of biochemical indicators for kidney function in *mdx* mice treated with PMO-GF. Data show no difference in the level of serum creatinine (Cr), urea and urinary kidney injury molecule-1 (KIM-1) in *mdx* mice treated with PMO-GF compared with untreated *mdx* and normal controls (n=6, error bars are $\pm$s.e.m; two-tailed t test, P>0.05), indicating no kidney injury trigged by the use of PMO-GF in *mdx* mice.
(**c**) Analysis of glucose level in *mdx* mice treated with PMO-GF. Data show no difference in the level of glucose in *mdx* mice treated with PMO-GF compared with untreated *mdx* and normal controls (n=6, error bars are $\pm$s.e.m; two-tailed t test, P>0.05).
(**d**) Hematoxylin & eosin staining of kidney (upper panel) and liver (lower panel) tissues sections from treated *mdx* mice, untreated *mdx* and *C57BL6* normal controls (scale bar=200 μm).
(**e**) Detection of CD3+ T lymphocytes and macrophage in diaphragm muscles from *mdx* mice treated with PMO-GF (scale bar=200 μm). Data show fewer $CD3^+$ T lymphocytes and macrophage in treated diaphragm muscle compared with untreated controls.
(**f**) Body-weight measurements of *mdx* mice treated with PMO-GF or PMO-S over 26 weeks. Data shows a steady body-weight increase and the same pattern of growth with both treatments as untreated *mdx* controls (n=6, error bars are $\pm$s.e.m). BW refers to Body Weight.

Supplementary Fig. 3

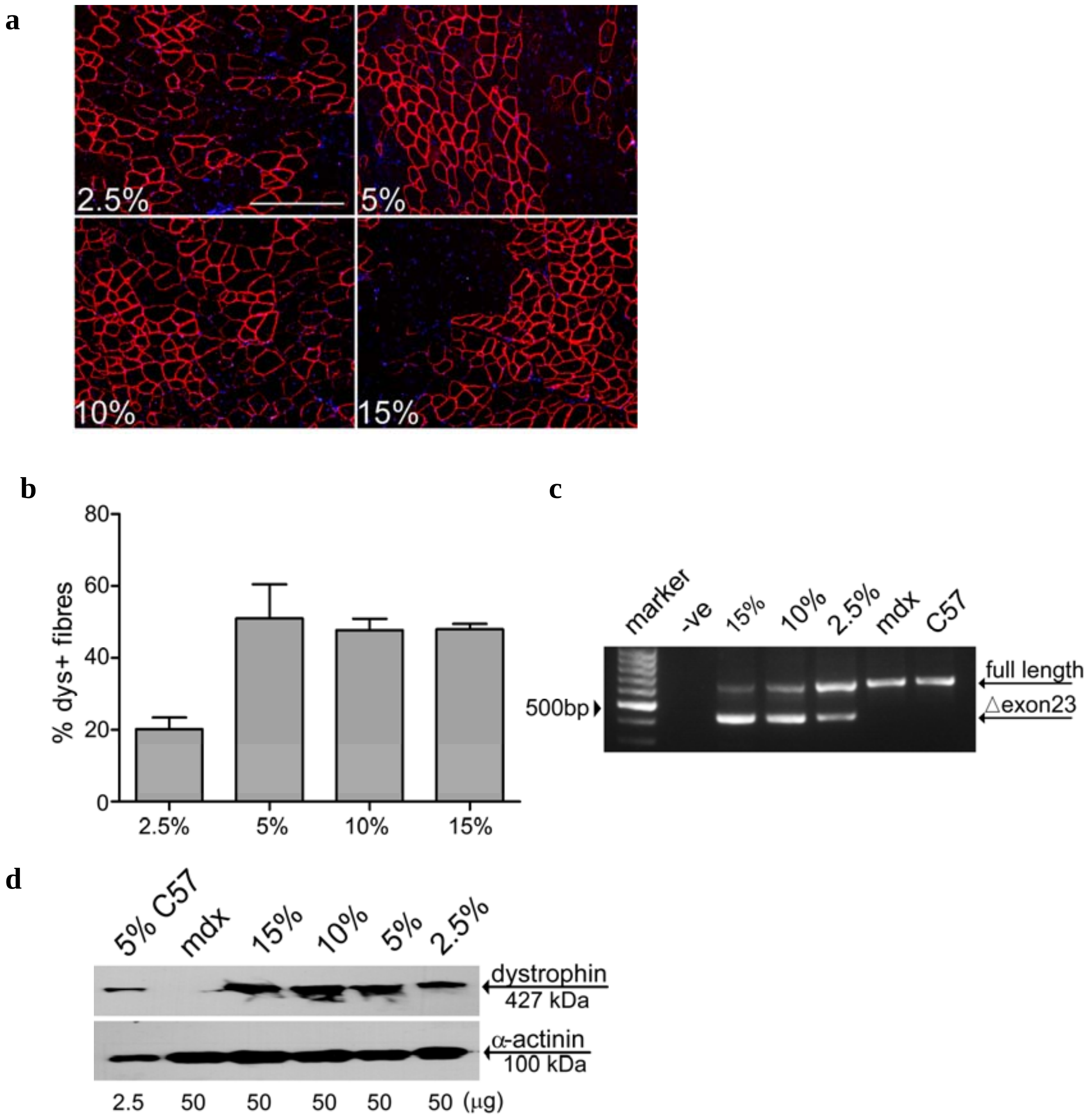

Supplementary Figure 3

Saturation effects of GF on increasing PMO exon skipping activity in *mdx* mice.

(**a**) Immunohistochemistry for dystrophin in *mdx* TA muscles following intramuscular injection 2 μg PMO formulated in different concentrations of GF in *mdx* mice (scale bar=200 μm).
(**b**) Quantitative evaluation of dystrophin-positive fibres in treated TA muscles. The data are presented as percent of dystrophin-positive fibres (n=6, error bars are ±s.e.m;).
(**c**) RT-PCR for detecting exon-skipping efficiency at the RNA level, which is shown by shorter exon-skipped bands (indicated by the numbered Δexon23 - exon 23 skipped).
(**d**) Western blot analysis of treated TA muscles. Protein loading was labelled below the image and TA muscles from *C57BL6* were used as normal controls. And α-actinin was used as the loading control.

Supplementary Fig. 4

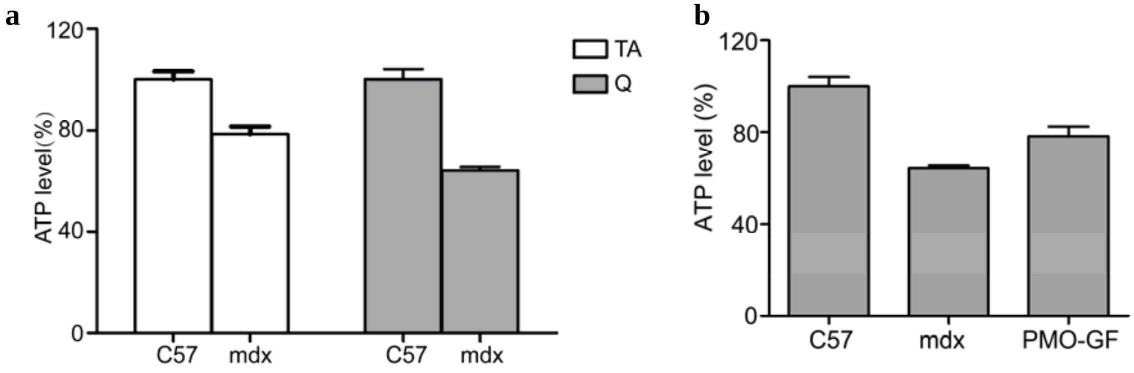

Supplementary Figure 4

Measurement of ATP levels in PMO-GF treated *mdx* and control mice.

(**a**) Basal levels of ATP in quadriceps (Q) and TA muscles from *mdx* and age-matched *C57BL6* mice were examined with CellTiter-Glo® Luminescent Cell Viability Assay kit (n=4, error bars are $\underline{+}$s.e.m).
(**b**) Levels of ATP in quadriceps from *mdx* mice treated with PMO-GF at the dose of 50 mg $kg^{-1}week^{-1}$ for 3 weeks followed by 50 mg $kg^{-1}month^{-1}$ for 5 months and compared with age-matched *mdx* and *C57BL6* mice (n=4, error bars are $\underline{+}$s.e.m).

Supplementary Fig. 5

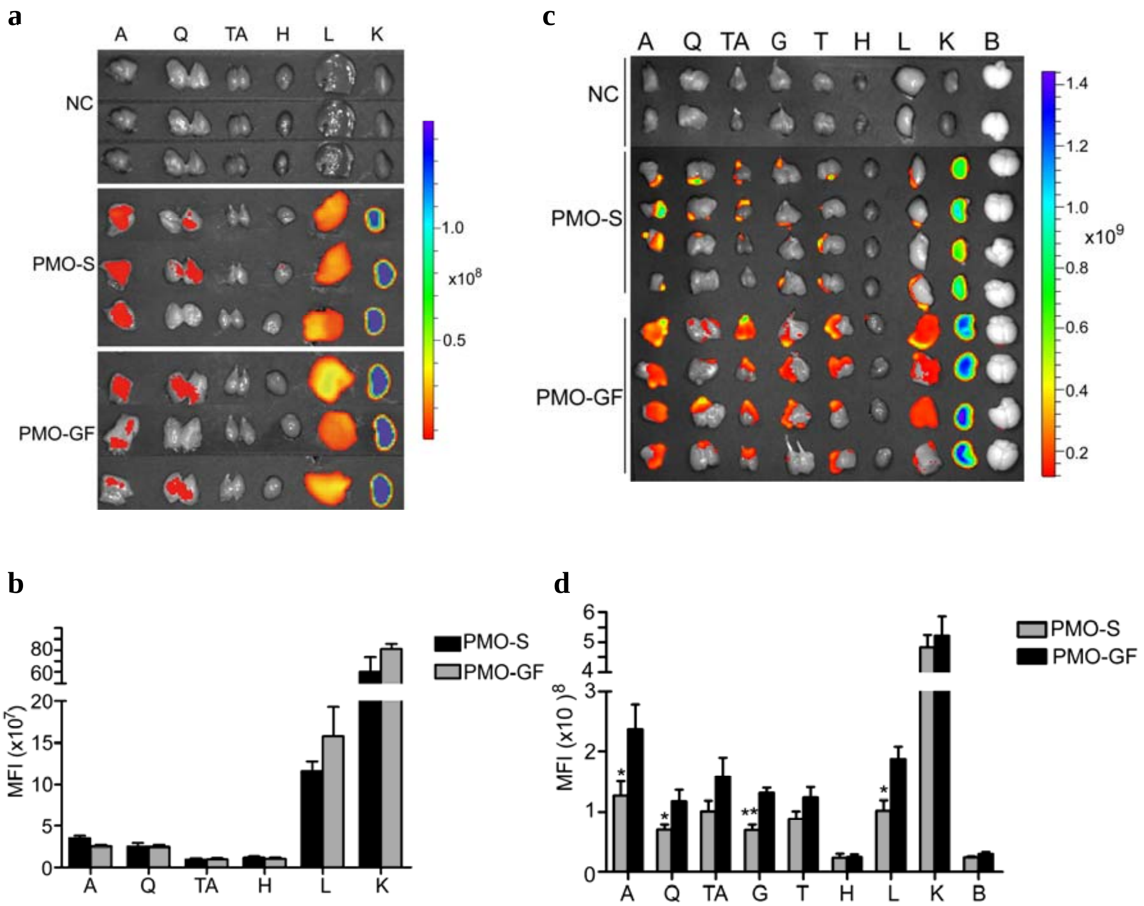

Supplementary Figure 5

Tissue distribution of lissamine-labelled PMO in *C57BL6* or *SOD1* mice.

(**a**) Measurement of tissue distribution of lissamine-labelled PMO in body-wide tissues with IVIS spectrum series in *C57BL6* mice. Lissamine-labelled PMO was injected into *C57BL6* mice intravenously at the dose of 25 mg kg^{-1} day^{-1} for 3 days and body-wide tissues were harvested 4 days after last injection.
(**b**) Quantitative analysis of mean fluorescence intensity in body-wide tissues from *C57BL6* mice treated with labelled PMO-GF or PMO-S (n=4, error bars are ±s.e.m).
(**c**) Measurement of tissue distribution of lissamine-labelled PMO in body-wide tissues with IVIS spectrum series in *SOD1* mice. A-abdominal muscle, Q-quadriceps, TA-tibialis anterior, G-gastrocnemius, T-triceps, H-heart, L-liver, K-kidney and B=brain.
(**d**) Quantitative analysis of mean fluorescence intensity in body-wide tissues from *SOD1* mice treated with labelled PMO-GF or PMO-S. Significant increase in mean fluorescence intensity was observed in Q, L (n=4, error bars are ±s.e.m; two-tailed t test, *$P<0.05$) and G (n=4, error bars are ±s.e.m; two-tailed t test, **$P=0.003$) from *SOD1* mice treated with PMO-GF compared to counterparts treated with PMO-S.

Supplementary Fig. 6

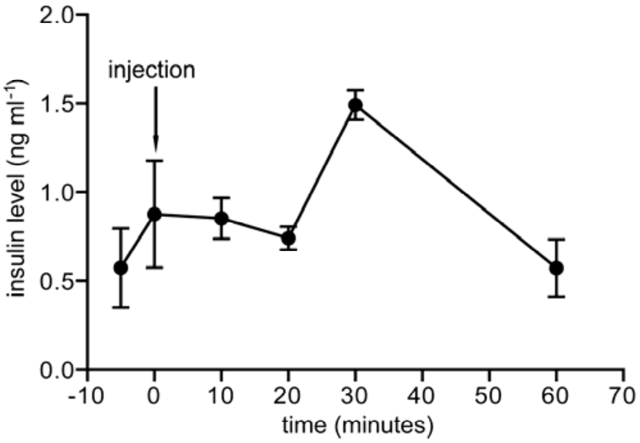

Supplementary Figure 6

Measurement of plasma insulin before and after GF administration in *mdx* mice.

GF (100 µl) was administered into adult *mdx* mice intravenously and plasma was harvested at different time-points including 5 min before injection and 5, 10, 20, 30, 60 min after injection (n=4, error bars are ±s.e.m).

Supplementary Fig. 7

Uncropped images of all Western blots.

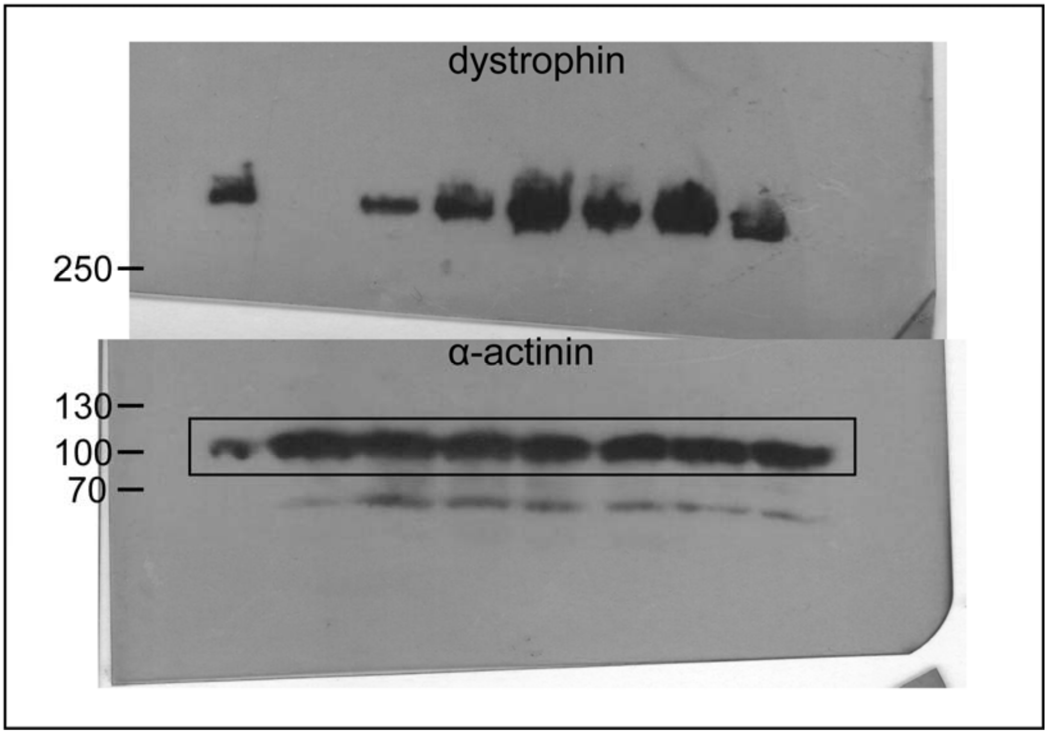

<u>Uncropped membranes for Figure 1b</u>

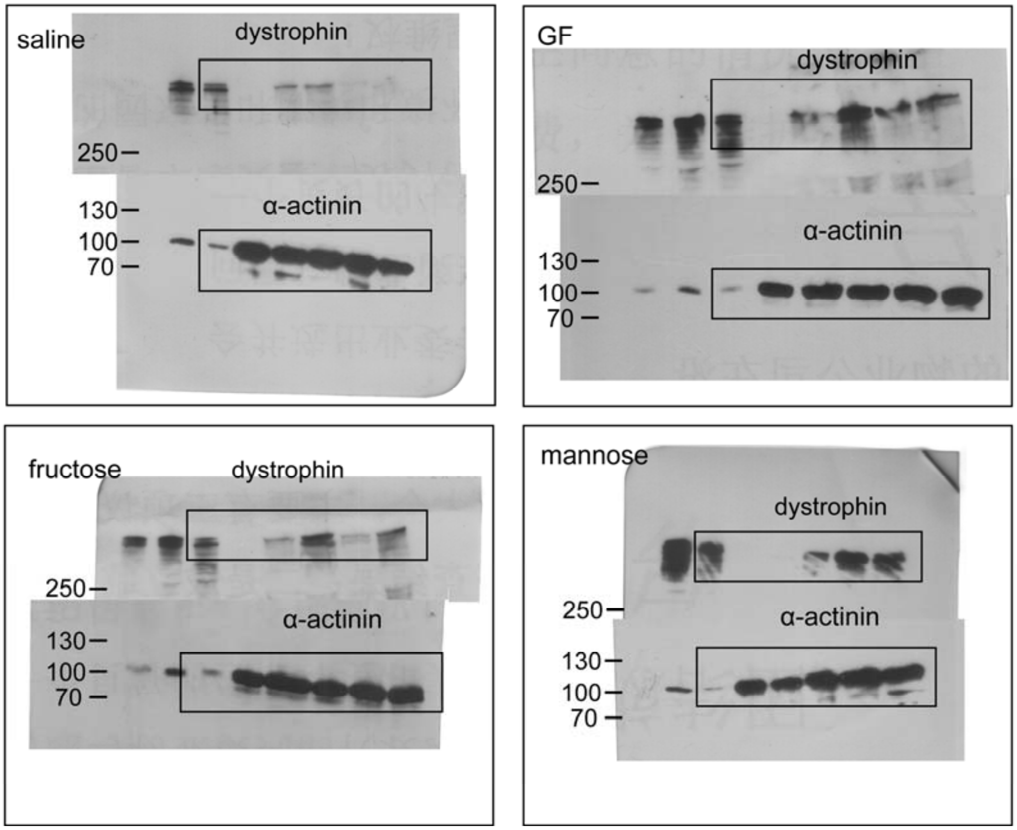

Uncropped membranes for Figure 1d

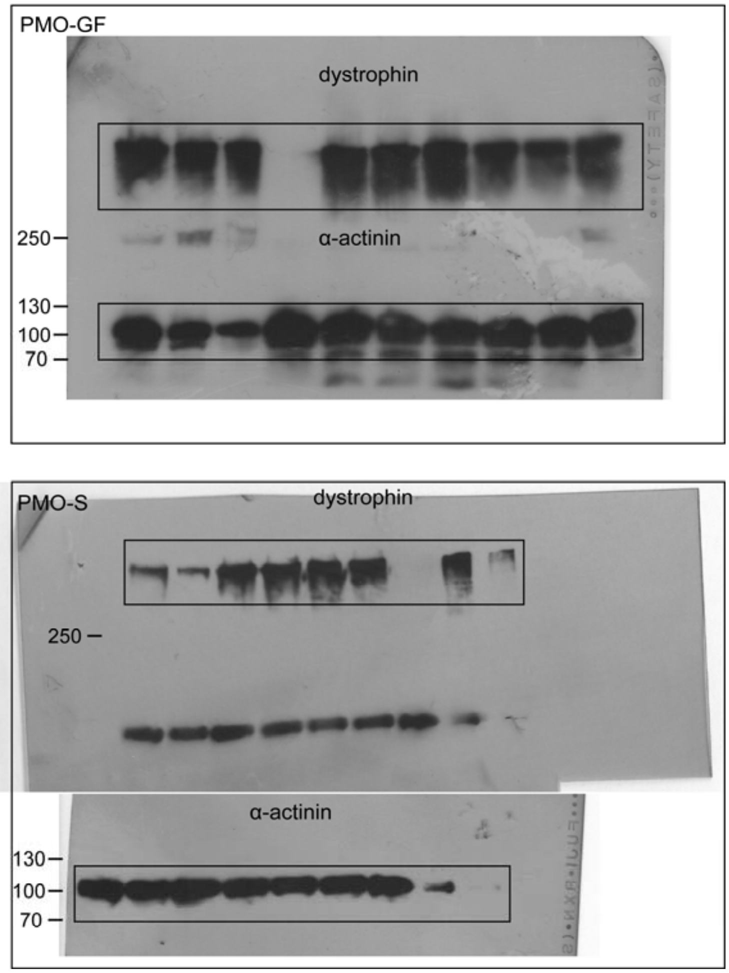

Uncropped membranes for Figure 2c

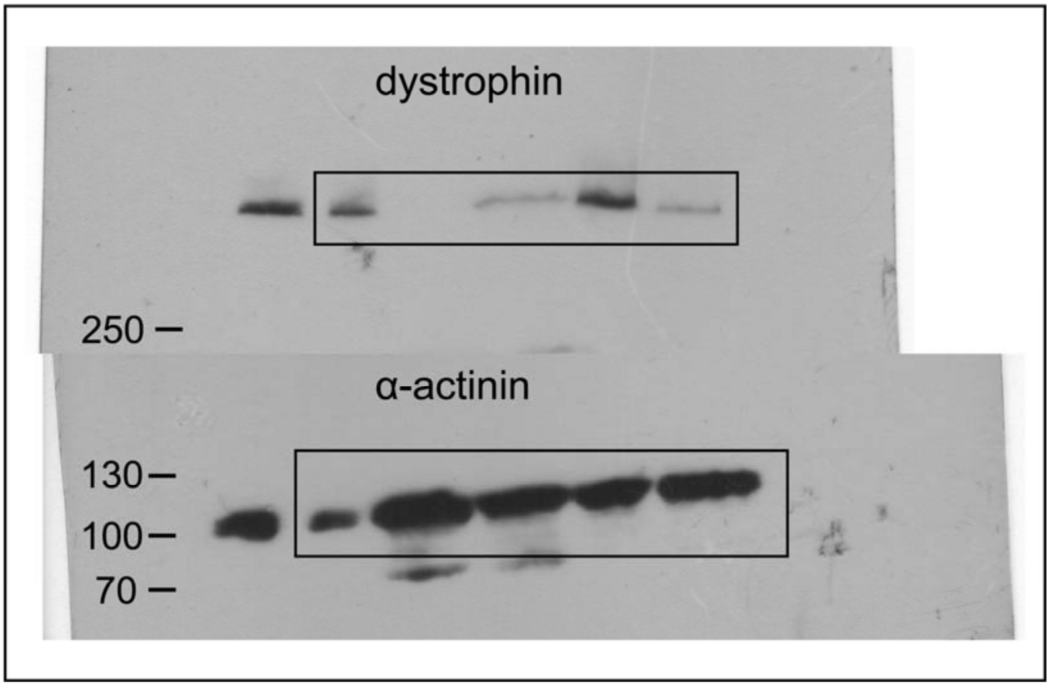

Uncropped membranes for Figure 3b

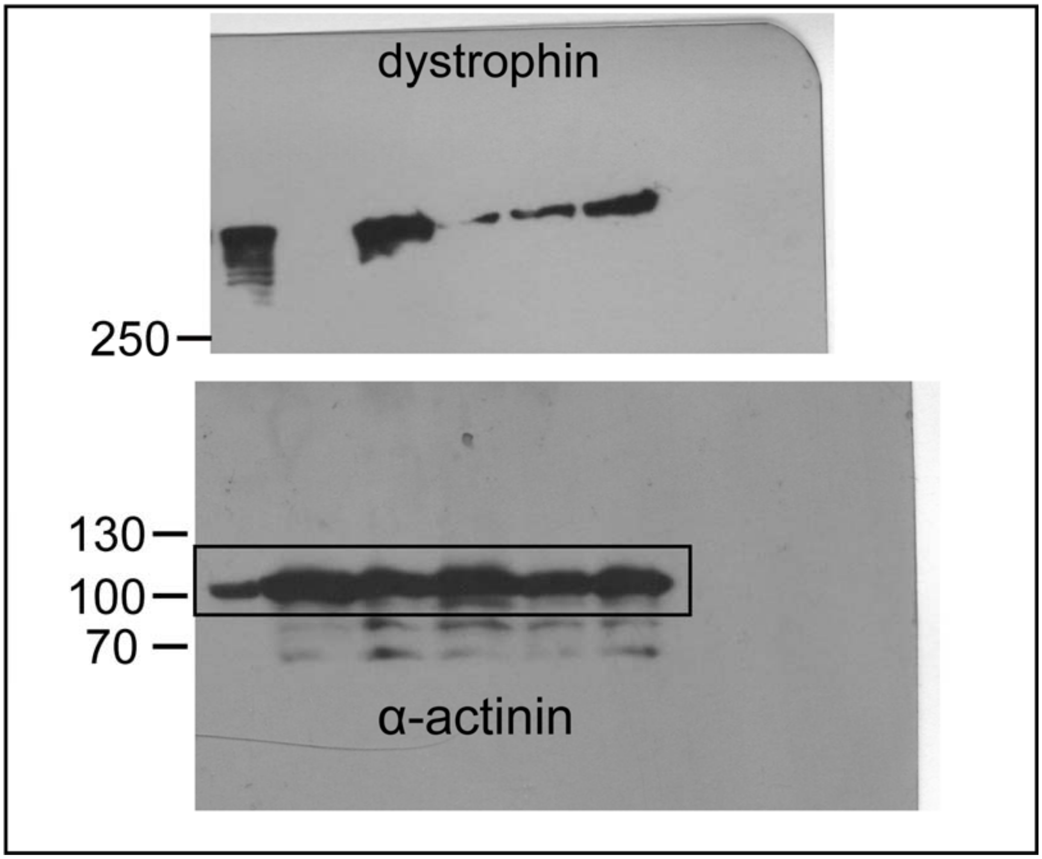

Uncropped membranes for Figure 3g

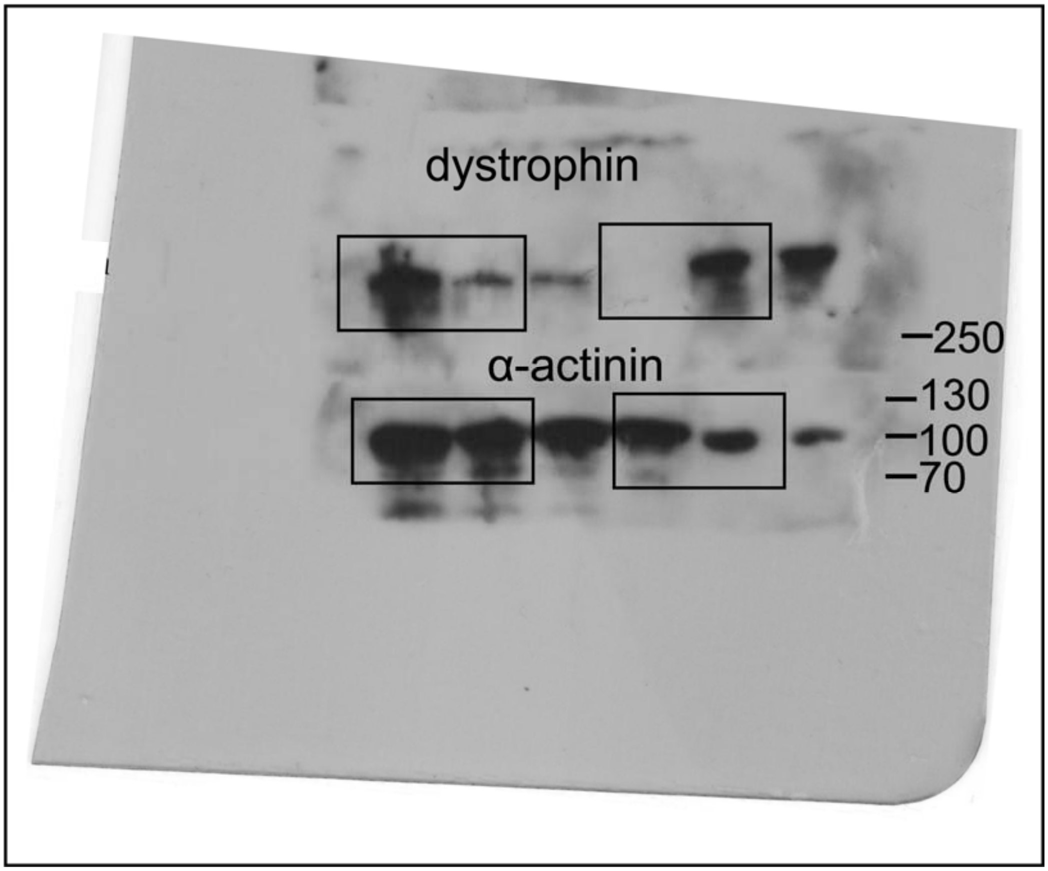

Uncropped membranes for Figure 3i

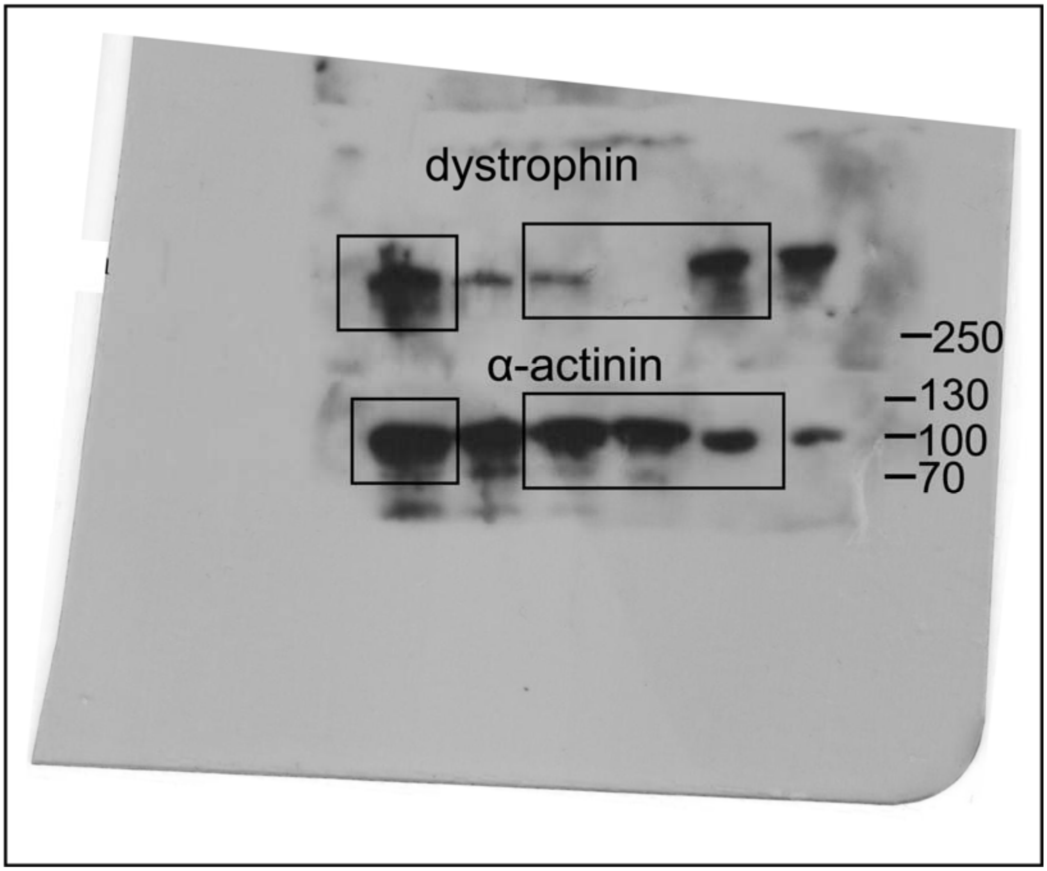

Uncropped membranes for Figure 4b

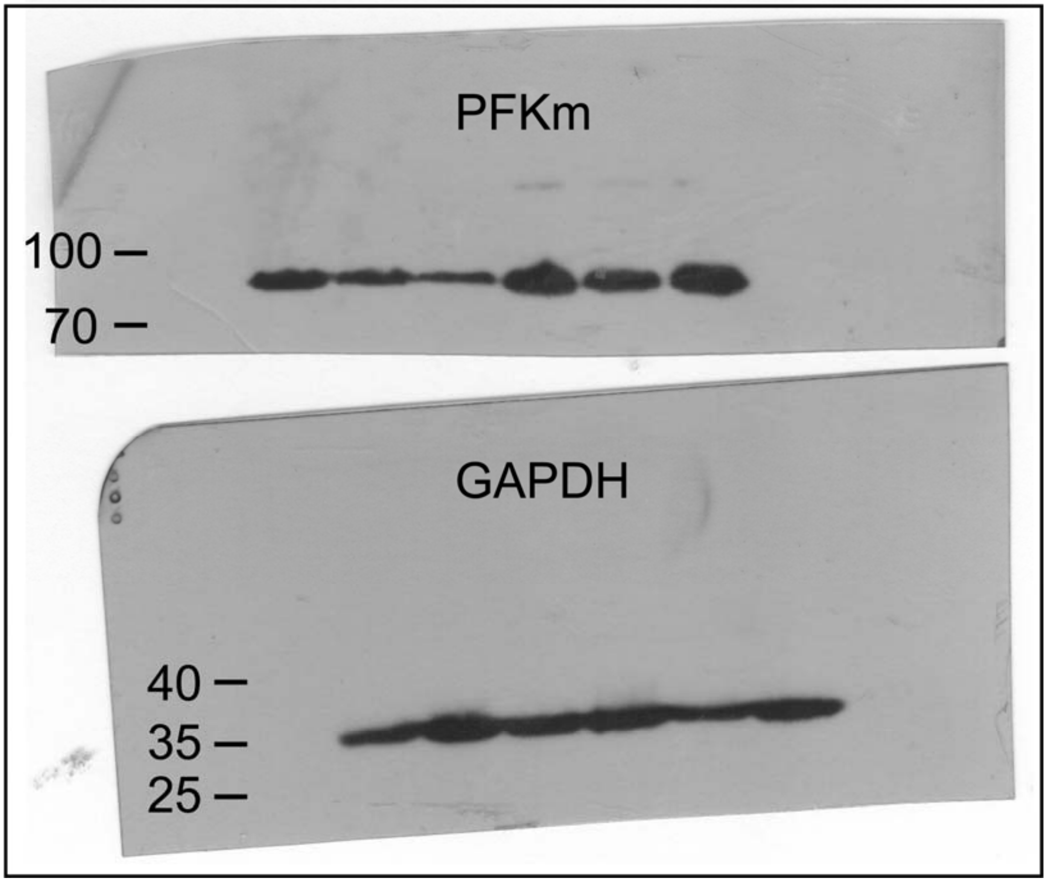

Uncropped membranes for Figure 4c

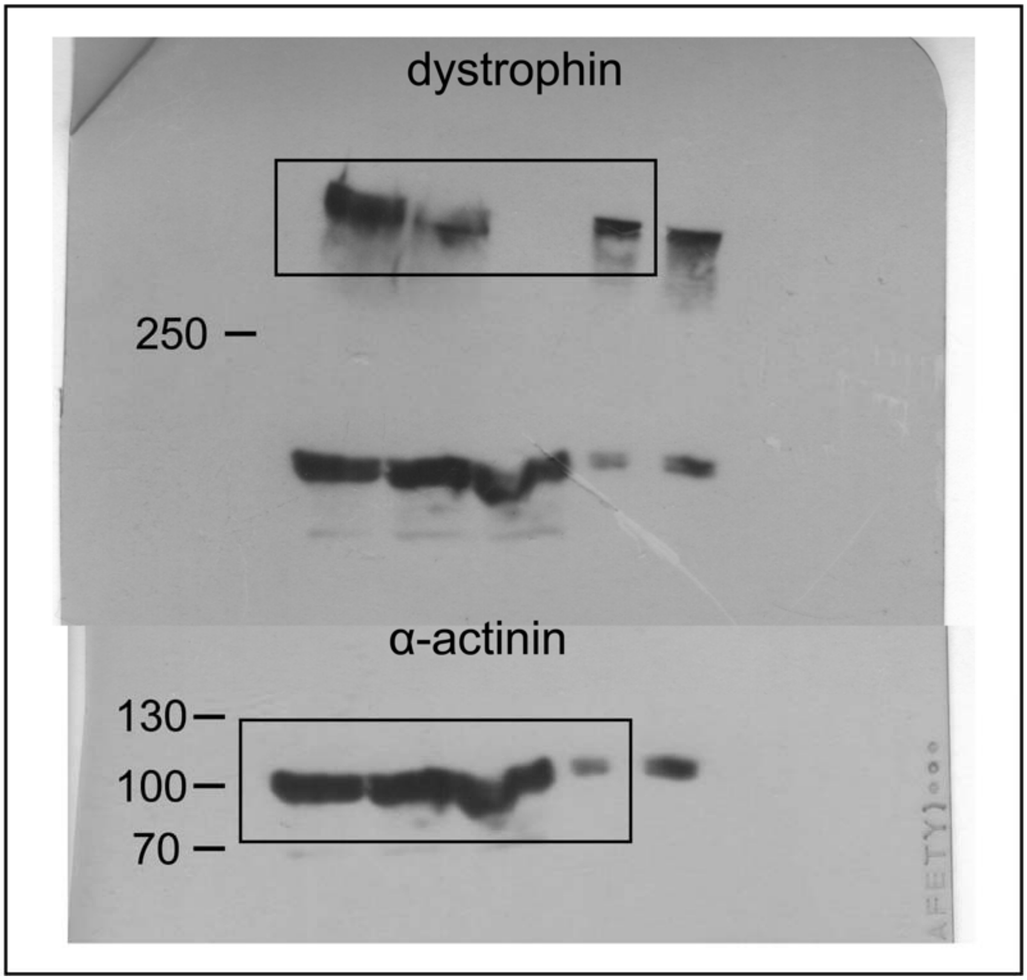

Uncropped membranes for Figure 4f

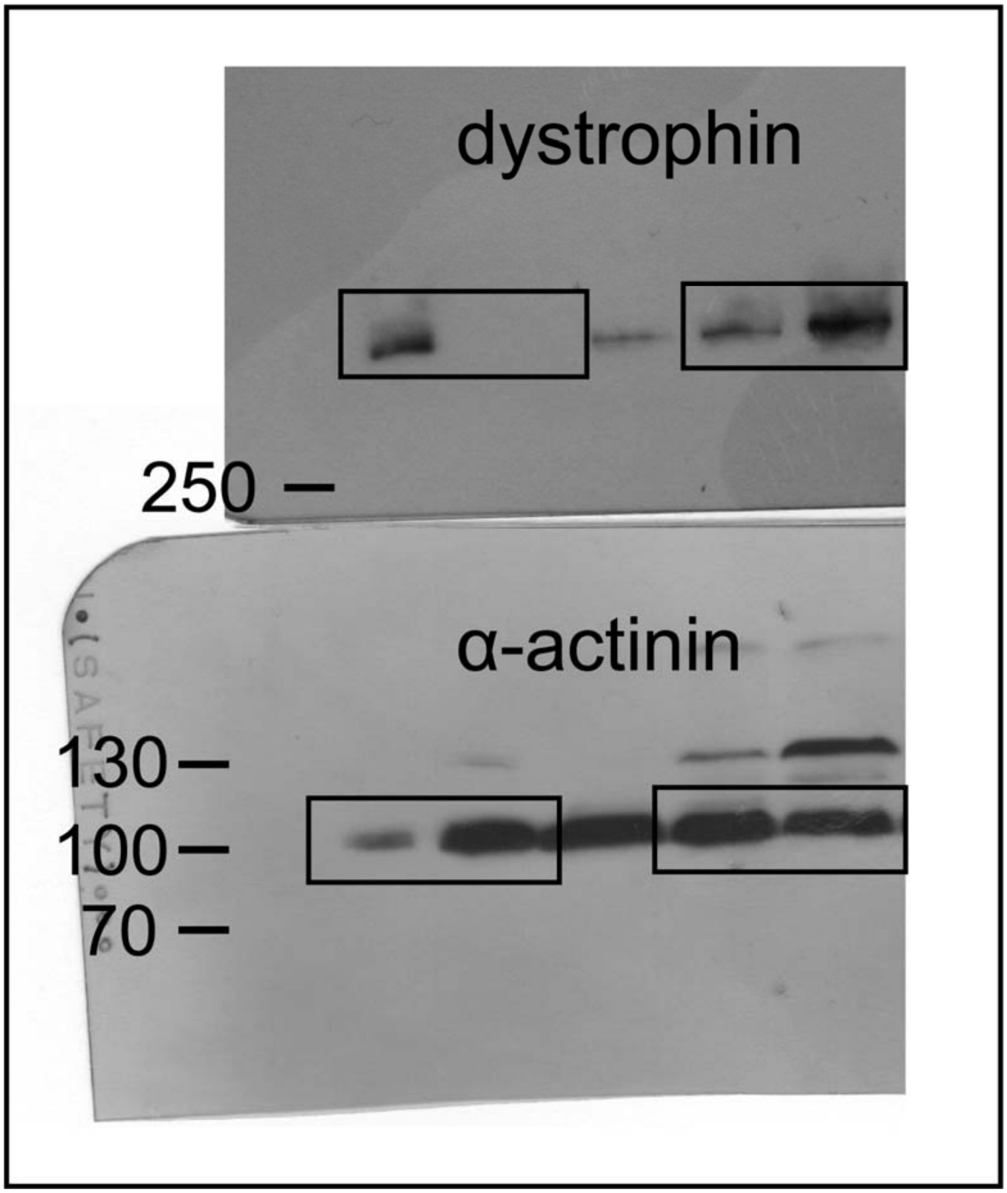

Uncropped membranes for Figure 4h

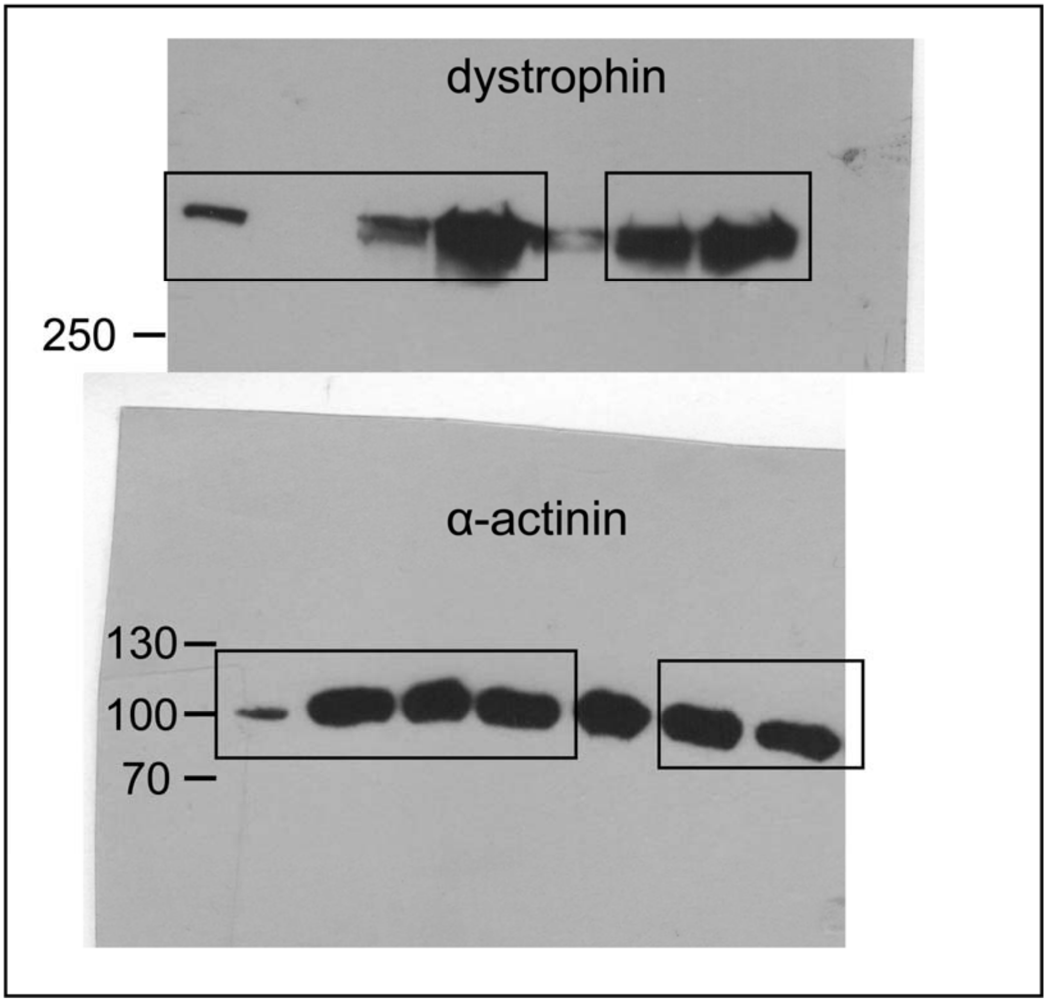

Uncropped membranes for Figure 5b

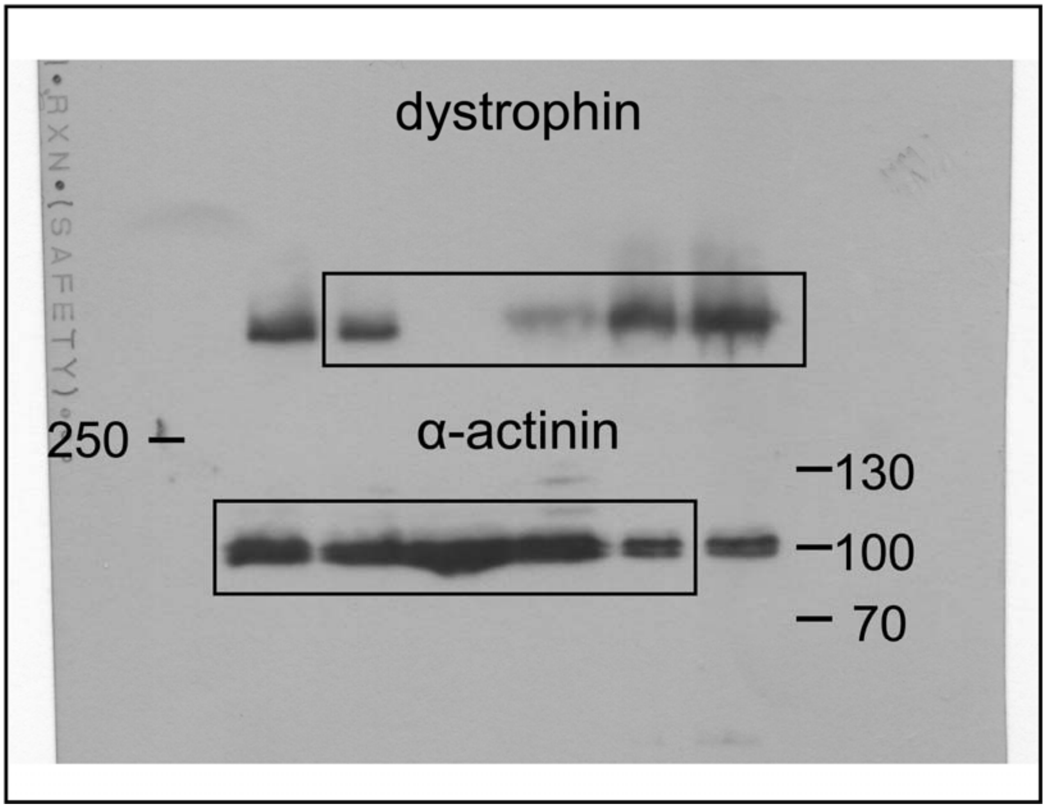

Uncropped membranes for Figure 5d

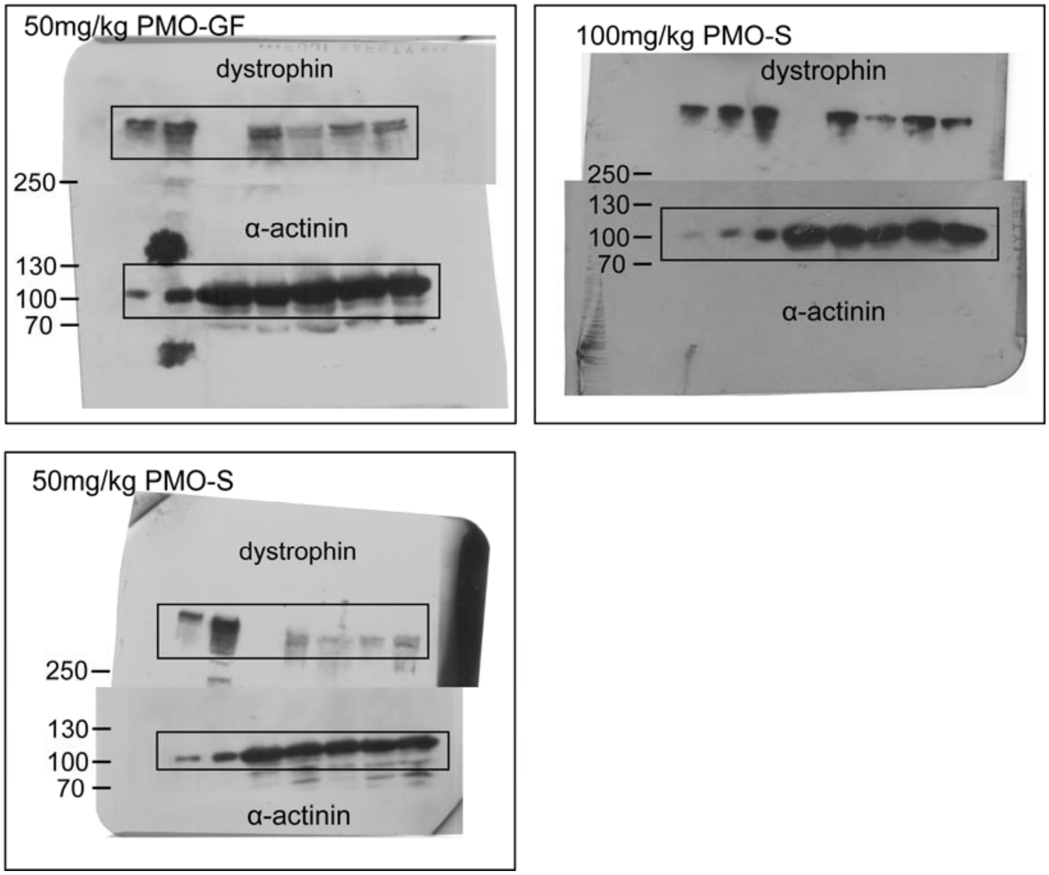

Uncropped membranes for Supplementary Figure 1b

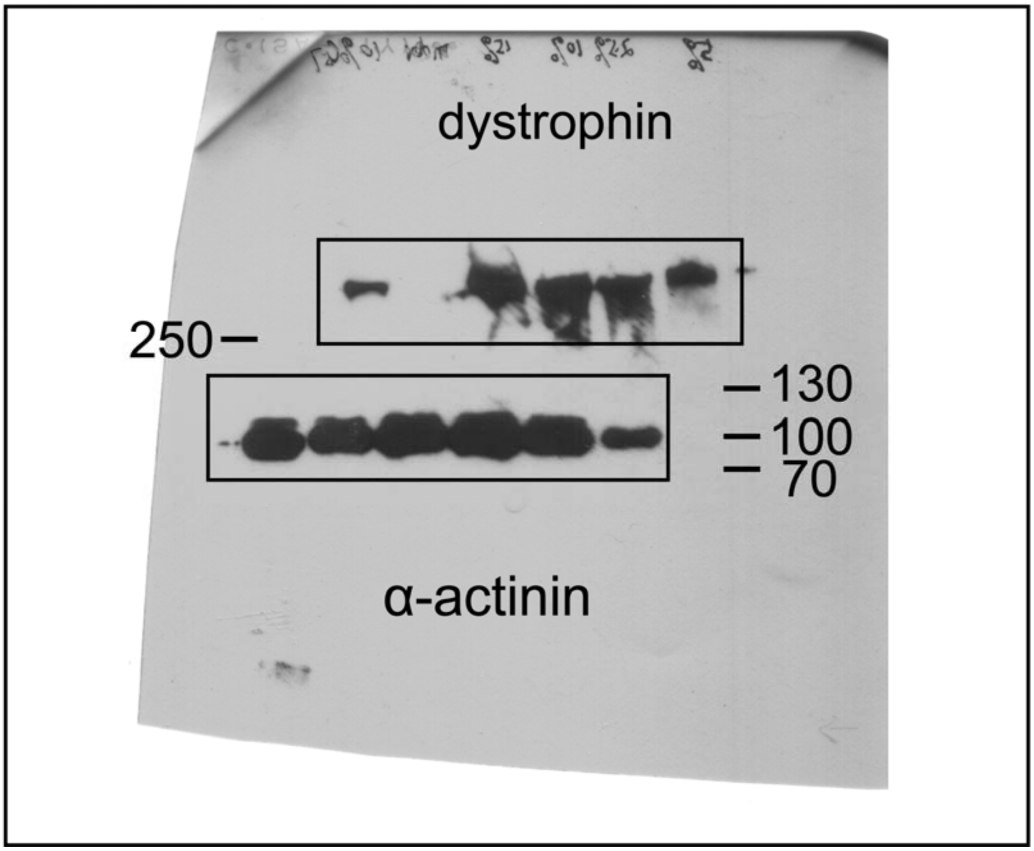

Uncropped membranes for Supplementary Figure 3d
